# Tyrosine-Phosphorylated Caveolin-1 Blocks Bacterial Uptake by Inducing Vav2-RhoA-Mediated Cytoskeletal Rearrangements

**DOI:** 10.1371/journal.pbio.1000457

**Published:** 2010-08-24

**Authors:** Jan Peter Boettcher, Marieluise Kirchner, Yuri Churin, Alexis Kaushansky, Malvika Pompaiah, Hans Thorn, Volker Brinkmann, Gavin MacBeath, Thomas F. Meyer

**Affiliations:** 1 Department of Molecular Biology, Max Planck Institute for Infection Biology, Berlin, Germany; 2 Department of Chemistry and Chemical Biology, Harvard University, Cambridge, Massachusetts, United States of America; University of Vermont, United States of America

## Abstract

Certain bacterial adhesins appear to promote a pathogen's extracellular lifestyle rather than its entry into host cells. However, little is known about the stimuli elicited upon such pathogen host-cell interactions. Here, we report that type IV pili (Tfp)-producing *Neisseria gonorrhoeae* (P^+^GC) induces an immediate recruitment of caveolin-1 (Cav1) in the host cell, which subsequently prevents bacterial internalization by triggering cytoskeletal rearrangements via downstream phosphotyrosine signaling. A broad and unbiased analysis of potential interaction partners for tyrosine-phosphorylated Cav1 revealed a direct interaction with the Rho-family guanine nucleotide exchange factor Vav2. Both Vav2 and its substrate, the small GTPase RhoA, were found to play a direct role in the Cav1-mediated prevention of bacterial uptake. Our findings, which have been extended to enteropathogenic *Escherichia coli*, highlight how Tfp-producing bacteria avoid host cell uptake. Further, our data establish a mechanistic link between Cav1 phosphorylation and pathogen-induced cytoskeleton reorganization and advance our understanding of caveolin function.

## Abstract 2

During the early stages of infection, *Neisseria gonorrhoeae* triggers a phosphotyrosine-dependent Cav1-Vav2-RhoA signaling cascade that promotes the pathogen's extracellular state.

## Author Summary

Like many bacterial pathogens, successful attachment of *Neisseria gonorrhoeae*—the causative agent of the sexually transmitted disease gonorrhoea—to its host cells depends on specialized structures on the bacterial surface called type IV pili (Tfp). Pathogen attachment induces changes within host cells that may facilitate and promote infection. In this study, we identify some of the earliest cellular signals elicited by *N. gonorrhoeae* during infection, which, in this case, prevent the organism from entering the cell precociously. After attachment to host cells the bacteria form microcolonies on the cell surface. Underneath these microcolonies, so-called cortical plaques form within the host cell—these contain the cytoskeleton protein actin and a range of signaling proteins. We show that *N. gonorrhoeae* recruits a host cell protein called caveolin-1 to the cell membrane where the bacteria are attached; here, caveloin-1 effectively impedes uptake of the bacteria by activating a signaling cascade that involves its phosphorylation on a tyrosine residue and subsequent interactions with proteins that regulate the cytoskeleton. Thus, these proteins play a pivotal role in maintaining *N. gonorrhoeae* in the extracellular milieu. By extrapolating our findings to another Tfp-producing bacterium, the enteropathogenic *Escherichia coli*, we argue that the establishment and maintenance of this extracellular state benefits certain pathogens by giving them time to express proteins required for subsequent steps of infection.

## Introduction

The primarily extracellular obligate human pathogen *Neisseria gonorrhoeae* (P^+^GC) is the causative agent of the sexually transmitted disease gonorrhoea, affecting over 60 million people every year worldwide [1]. It is a type IV pili **(**Tfp)-producing bacteria that colonizes mucosal epithelia of the human urogenital tract [2]. Tfp are proteinaceous filaments that play a crucial role in pathogenesis by mediating the initial attachment to host cell receptors and are expressed on the surface of a variety of bacterial pathogens such as Gram-negative *N. meningitidis*, *Pseudomonas aeruginosa*, and *enteropathogenic E. coli* (EPEC) as well as Gram-positive *Streptococcus sanguis* and *Clostridium perfringens* [3]. A growing body of evidence suggests that adhesins such as Tfp are key pathogenesis factors facilitating not only attachment but soliciting the necessary host cell cytoskeletal rearrangements and signaling cascades that promote an extracellular lifestyle [4]. Tfp-expression and cytoskeletal remodeling allows *N. meningitidis* to resist shear stress possibly encountered in the bloodstream [5], and mechanical forces generated by pilus retraction of P^+^GC lead to cytoprotection [6]. However, details of the elicited signaling cascades within the host cell upon attachment of *Neisseria* remain patchy and require clarification.

The early stages of infection with P^+^GC are characterized by Tfp-mediated attachment to host cells [7]. This is followed by retraction of pili in a force-generating depolymerization process [8] and formation of microcolonies on the surface of host epithelial cells [2]. Cortical actin and various signal transducing proteins are then recruited to the site of bacterial attachment [9]. As infection proceeds, the phase-variable opacity associated (Opa) proteins are expressed, allowing occasional entry and transcytosis of individual bacteria through epithelial cells to reach underlying tissues [10]. Several signaling proteins that are recruited to P^+^GC microcolonies have also been found to be associated with lipid rafts and caveolae, cholesterol-enriched microdomains of cell membranes [11], suggesting that these or associated proteins play an essential role in this initial infection step. The major structural protein of plasma membrane caveolae, caveolin-1 (Cav1), is also known to localize to subcellular compartments and to the cytoplasm [12]. Cav1 has been shown to inhibit signal transduction by binding to numerous target proteins with its scaffolding domain [12], but it can also promote signaling events through phosphorylation on tyrosine 14 (Tyr14) [13],[14]. We speculated therefore that Cav1 could play an important role during P^+^GC infection. Here we provide evidence that during the early stages of infection, P^+^GC triggers a phosphotyrosine-dependent Cav1-Vav2-RhoA signaling cascade that elicits cytoskeletal rearrangements and effectively impedes bacterial uptake into host cells.

## Results/Discussion

**Figure 1:**
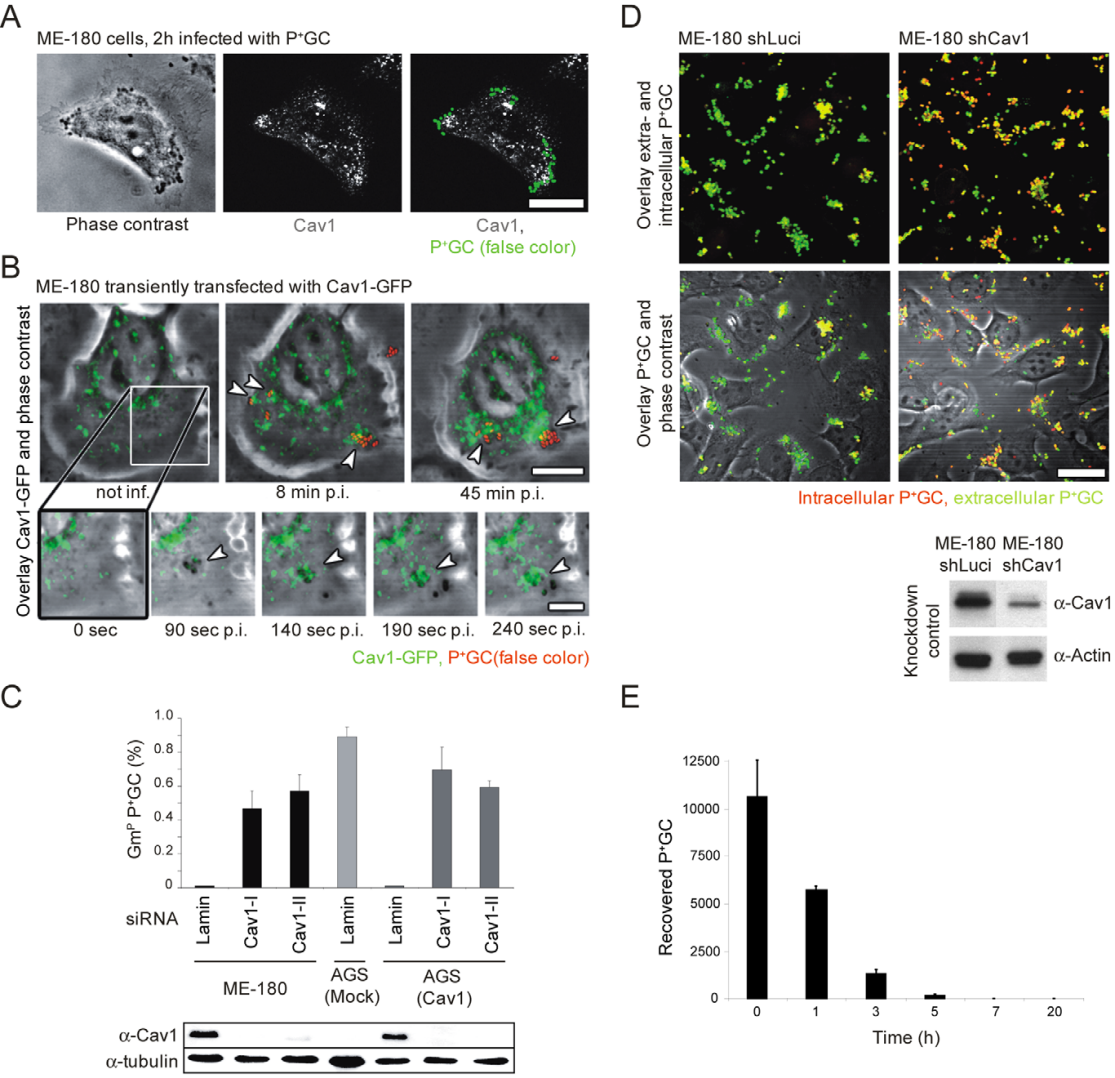
Expression and recruitment of Cav1 prevents internalization of P^**+**^GC by host cells. (A) Recruitment of endogenous Cav1 (white, middle panel) to attached P^+^GC (green, right panel) in ME-180 cells 2 h post-infection. (B) Excerpts of Movie 1: Cav1-GFP (green) is recruited within seconds to microcolonies and individually attached P^+^GC (red) in ME-180 cells (lower panels). Recruitment continues as infection proceeds (upper panels). Attached P^+^GC are indicated by arrows. (C) Knockdown of Cav1 in ME-180 cells and in Cav1 expressing AGS cells (AGS-Cav1) results in P^+^GC internalization. Cav1 expression was downregulated by transfection of two different siRNAs (Cav1-I or Cav1-II) in ME-180 and AGS-Cav1 cells using lamin A/C as a control siRNA. Gentamicin protection assays were performed 2 h post-infection (upper panel). Experiments were performed in triplicate. Data are mean ± standard deviation. Cav1 knockdown efficiency was confirmed by Western blot analysis (lower panel). (D) shRNA-mediated downregulation of Cav1 in ME-180 cells results in P^+^GC internalization. Intracellular bacteria (red) are detected in ME-180 shCav1 cells (upper right panels), whereas only extracellular bacteria (yellow-green) are detected in ME-180 shLuciferase control cells (upper left panels). Efficiency of Cav1 knockdown in ME-180 cells after lentiviral transduction of Luciferase (control) or Cav1 shRNA constructs (lower panel). Scale bar: 20 µm. (E) Numbers of viable intracellular P^+^GC in AGS cells decrease rapidly over time. Infected cells were initially treated with gentamicin for 2 h, then further incubated in gentamicin and serum-free medium and lysed at indicated time points. Experiments were performed in triplicate. Data are mean ± standard deviation.

To assess the role of Cav1 in the Tfp-mediated binding of P^+^GC to host cells, we began by monitoring the cellular localization of Cav1 in ME-180 cells, a human epidermoid carcinoma cell line, immediately following infection. We found that endogenous Cav1 localized close to P^+^GC microcolonies after 2 h of infection (Figure 1A). Using live-cell imaging, we likewise observed a substantial accumulation of Cav1-GFP at sites of bacterial infection, which was induced even by single diplococci and within seconds after P^+^GC attachment (Figure 1B, lower panel). Cav1-GFP recruitment occurred throughout the early stages of infection, resulting ultimately in a conspicuous accumulation of the protein (Figure 1B, upper panel and Video S1). By contrast, using a non-piliated isogenic GC strain (P^−^ Opa57^+^GC is a non-piliated isogenic strain of P^+^GC that produces an Opa57 adhesin specific for CEACAM receptors), we found no recruitment of endogenous Cav1 in ME-180 cells (Figure S1A). Interestingly, despite the observed recruitment of Cav1 after P^+^GC infection, microarray and Western blot analysis failed to detect any increase in Cav1 expression (unpublished data). This rather points to a cellular Cav1 reorganization leading to Cav1-accumulation than de novo synthesis.

The functional role of Cav1 in bacterial infection was then explored by downregulating Cav1 levels in ME-180 cells using RNA interference (RNAi). Reduced Cav1 levels resulted in efficient internalization of P^+^GC by ME-180 cells, as demonstrated by gentamicin protection assays (Figure 1C) and confocal microscopy (Figure 1D). Next, we turned to the human gastric carcinoma cell line AGS, which, like many other malignant cell lines, is devoid of detectable levels of Cav1 [15]. Stably transfected cell lines, AGS-Mock and AGS-Cav1, were infected with P^+^GC for 2 h and bacterial uptake was monitored by gentamicin protection assay. In contrast to mock-transfected cells, Cav1-expressing AGS cells inhibited P^+^GC internalization (Figure 1C). As before, siRNA-mediated downregulation of Cav1 in AGS-Cav1 cells restored bacterial uptake (Figure 1C). The total number of cell-associated bacteria was similar in the AGS and ME-180 cells and was unaffected by Cav1 expression. In addition, inhibition of bacterial uptake was Tfp-specific, as Opa-mediated bacterial uptake remained unaltered by Cav1 expression (Figure S1B). Finally, bacterial uptake was independent of pilus retraction, as indicated by the use of an isogenic, non-retractile PilT-deficient GC mutant (Figure S1C). Interestingly, in Cav1-negative AGS cells the observed epithelial cell entry resulted in a drastic decrease over time in the viability of internalized bacteria (Figure 1E). Taken together, these results demonstrate that Cav1 plays a pivotal role in preventing internalization of P^+^GC by host epithelial cells, thus promoting bacterial survival.

To broaden our findings, we also investigated Cav1 recruitment in EPEC infection, as initial adherence of EPEC to intestinal epithelial cells is conducted by type IV bundle-forming pili [16]. To avoid super-imposition with the actin-recruiting function of the EPEC type III secretion system (TTSS; [17]), we used a TTSS deficient mutant that still expressed Tfp. Interestingly, we found that the infection of epithelial cells by EPEC also induced an accumulation of Cav1 beneath the bacteria, similar to our observations with P^+^GC (Figure S2A). Most importantly, Tfp-producing EPEC entered Cav1 deficient AGS cells more rapidly compared to cells producing Cav1 (Figure S2B). This pronounced effect was even observed in the presence of the EPEC TTSS, thus emphasizing a generalized role of Cav1 in blocking cell entry of Tfp-producing bacteria. Together, our data suggest that Cav1 accumulation is a Tfp-specific and immediate cellular response to bacterial attachment that occurs throughout the early stages of infection.

**Figure 2:**
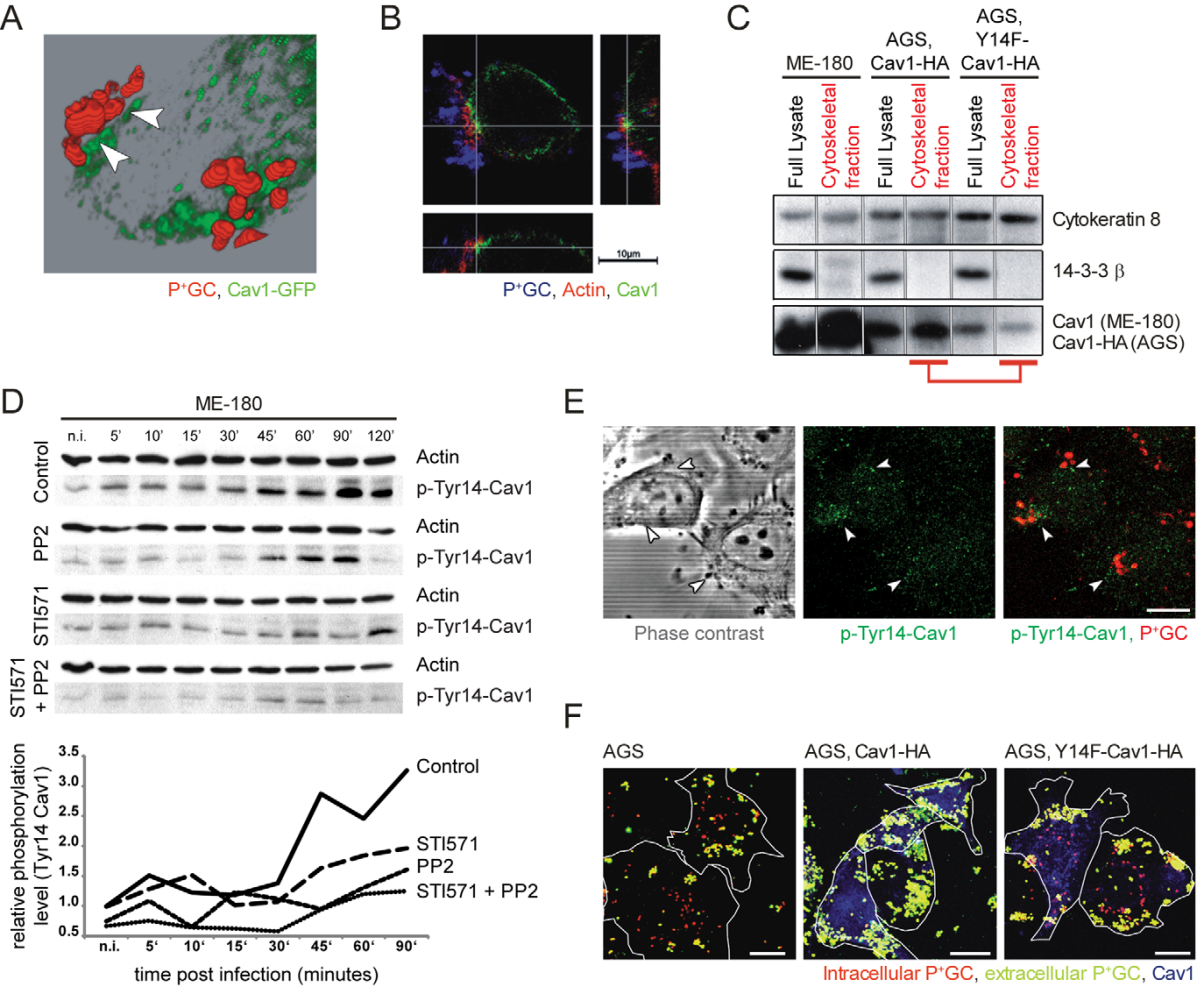
Tyrosine-phosphorylation of Cav1 is required for Cav1 association with the cytoskeleton and to prevent internalization of P^**+**^GC by host cells. (A) 3D reconstruction of confocal images depicting Cav1-GFP (green) recruitment in the host cell 2 h post-infection with P^+^GC (red). Gaps between P^+^GC and Cav1 are indicated by arrows. (B) Horizontal and vertical confocal image sections show F-actin (red) localization between bacteria (blue) and endogenous Cav1 (green) in infected ME-180 cells. Scale bar: 10 µm. (C) Interaction of Cav1 with cytoskeletal components depends on Cav1 phosphorylation. Fractionation of transfected AGS cells reveals a strong association of wild-type Cav1 (Cav1-HA), but not the phosphorylation-deficient mutant (Y14F-Cav1-HA), with cytoskeletal components (lower panel, red marking). Quantification of band intensities revealed a marked decrease (55%) in the association of Y14F-Cav1-HA with the cytoskeleton as compared to Cav1-HA. Endogenous Cav1 also associates with cytoskeletal components in ME-180 cells (lower panel). Cytokeratin 8 serves as a control for the correct localization of cytoskeleton-associated proteins (upper panel). 14-3-3β serves as a control for complete removal of cytoplasm-associated proteins from the cytoskeletal components fraction (middle panel). Full lysate rows serve as loading and protein expression controls. (D) Cav1 phosphorylation during P^+^GC infection depends on Abl and Src kinases. Western blot analysis of phospho-Tyr14-Cav1 levels (upper panels) and quantification of data (lower panel) show elevated Cav1 phosphorylation starting 5 min after infection. Stimulation of phosphorylation by P^+^GC is reduced in Src-inhibitor PP2 and Abl-inhibitor STI571-treated cells (both 10 µM). (E) Recruitment of phospho-Tyr14-Cav1 (green, middle panel, white arrows) to attached P^+^GC (red, right panel) in ME-180 cells 2 h post-infection. Scale bar: 10 µm. (F) Bacterial uptake is observed in Cav1-negative, AGS cells (left panel) but not in wild-type Cav1-transfected AGS cells (middle panel). By contrast, transfection of the phosphorylation-deficient mutant, Y14F-Cav1, does not impede bacterial uptake (right panel). Bacterial infection did not change the localization of Y14F-Cav1 in cells (right panel). Intracellular bacteria appear in red, extracellular bacteria in yellow-green, and Cav1 in blue. Cellular borders are represented as white outlines. Scale bars: 20 µm. Data in A–F are representative of three independent experiments.

Cav1 has also been shown to localize to non-caveolar cellular regions, where it participates in transport of signaling proteins via phosphorylation on Tyr14 [18]–[20]. We hypothesized that Cav1 recruited to P^+^GC microcolonies localizes outside caveolae and plays a role in protein trafficking or signaling. To address this question, we mapped the location of Cav1 in infected host cells using 3D-reconstruction of confocal images and immunogold staining. We found that Cav1 accumulates in the vicinity of P^+^GC but not directly at the plasma membrane (Figure 2A and Figure S3). Analysis of horizontal sections of confocal image stacks revealed F-actin structures in infected cells localized between the plasma membrane and endogenous Cav1 (Figure 2B). This is consistent with previous observations on the assembly of F-actin structures in epithelial cells following the attachment of P^+^GC [9], corroborating an association between F-actin and Cav1. Indeed, treatment of ME-180 cells with either cytochalasin D (Cyt D) or latrunculin A (Lat A), which disrupt actin filaments, prevented Cav1 accumulation (Figure S4A) and induced bacterial internalization as shown by confocal imaging (unpublished data) and gentamicin protection assays (Figure S4B). Thus, both recruitment of Cav1 and inhibition of bacterial internalization require a functional actin cytoskeleton.

Cav1 has previously been reported to bind cytoskeletal components such as the actin-crosslinking protein filamin and intermediate filaments [21],[22]. Moreover, relocation of Cav1 to the caveolae-free front of migrating cells requires its distribution along cytoskeletal structures and is dependent on the presence of Tyr14 [23]. To determine if the Cav1-cytoskeleton association observed here was dependent on Tyr14 of Cav1, we purified the cytoskeletal fraction from AGS cells that had been transfected either with an epitope-tagged version of wild-type Cav1 (Cav1-HA) or with a phosphorylation-defective mutant (Y14F-Cav1-HA). Similar to endogenous Cav1 in ME-180 cells, Cav1-HA was completely recovered from the cytoskeletal fraction of transfected AGS cells, whereas only 45% of the total Y14F-Cav1-HA was detected in this fraction (Figure 2C). However, after P^+^GC infection no significant differences in the Cav1-cytoskeleton association were observed expressing either Cav1 construct (unpublished data). Thus, it is likely that phosphorylation of Cav1 on Tyr14 promotes its association with the cytoskeleton.

To understand the importance of Cav1 phosphorylation during infection with P^+^GC, we monitored the phosphorylation status of Cav1 during infection (2 h) of serum starved ME-180 cells. In addition, we blocked Src kinases and Abl kinases using chemical inhibitors previously reported to phosphorylate Cav1 [24],[25]. The Src family kinase inhibitor PP2 [26] and Abl tyrosine kinase inhibitor STI571/Imatinib [27] were added (10 µM each), individually and in combination, 1 h prior to infection. Western blot analysis of phospho-Tyr14-Cav1 levels (Figure 2D, upper panels) and quantification of data (Figure 2D, lower panel) showed P^+^GC were able to elicit Cav1 phosphorylation at Tyr14. In control-treated ME-180 cells, Cav1 phosphorylation levels were increased 1.5-fold after 5 min of infection. Phosphorylation levels then reached a plateau phase before rising again after 30 min, increasing up to 4-fold after 90 min of infection. In accordance with previous reports [24],[25], this phosphorylation depended on active Src- and Abl-kinases. Compared to untreated control cells, stimulation of phosphorylation by P^+^GC in PP2- and STI571-treated cells was markedly reduced during the whole infection period. Strikingly, ME-180 cells treated with both inhibitors exhibited minimal levels of phosphorylation and stimulation by P^+^GC was negligible. Next, we determined the cellular localization of phospho-Tyr14-Cav1 using confocal imaging (Figure 2E). Despite the low levels of immunostained phosphorylated Cav1, phospho-Tyr14-Cav1 was detected in the vicinity of attached P^+^GC, as observed previously with non-phosphorylated Cav1, suggesting a direct link between P^+^GC infection and Cav1 phosphorylation. Hence, we infected AGS cells expressing either wild-type Cav1-HA or Y14F-Cav1-HA with P^+^GC. In contrast to wild-type Cav1, Y14F-Cav1 was not recruited to bacterial attachment sites and did not impede internalization (Figure 2F), strongly suggesting that Cav1 phosphorylation at Tyr14 is induced or enhanced by P^+^GC, enabling Cav1 recruitment, Cav1-mediated prevention of bacterial uptake, and a strong association of Cav1 with the cytoskeleton. Taken together, our data suggest that Cav1 phosphorylation plays a role in downstream signaling, linking Cav1 with cytoskeletal rearrangements.

**Figure 3:**
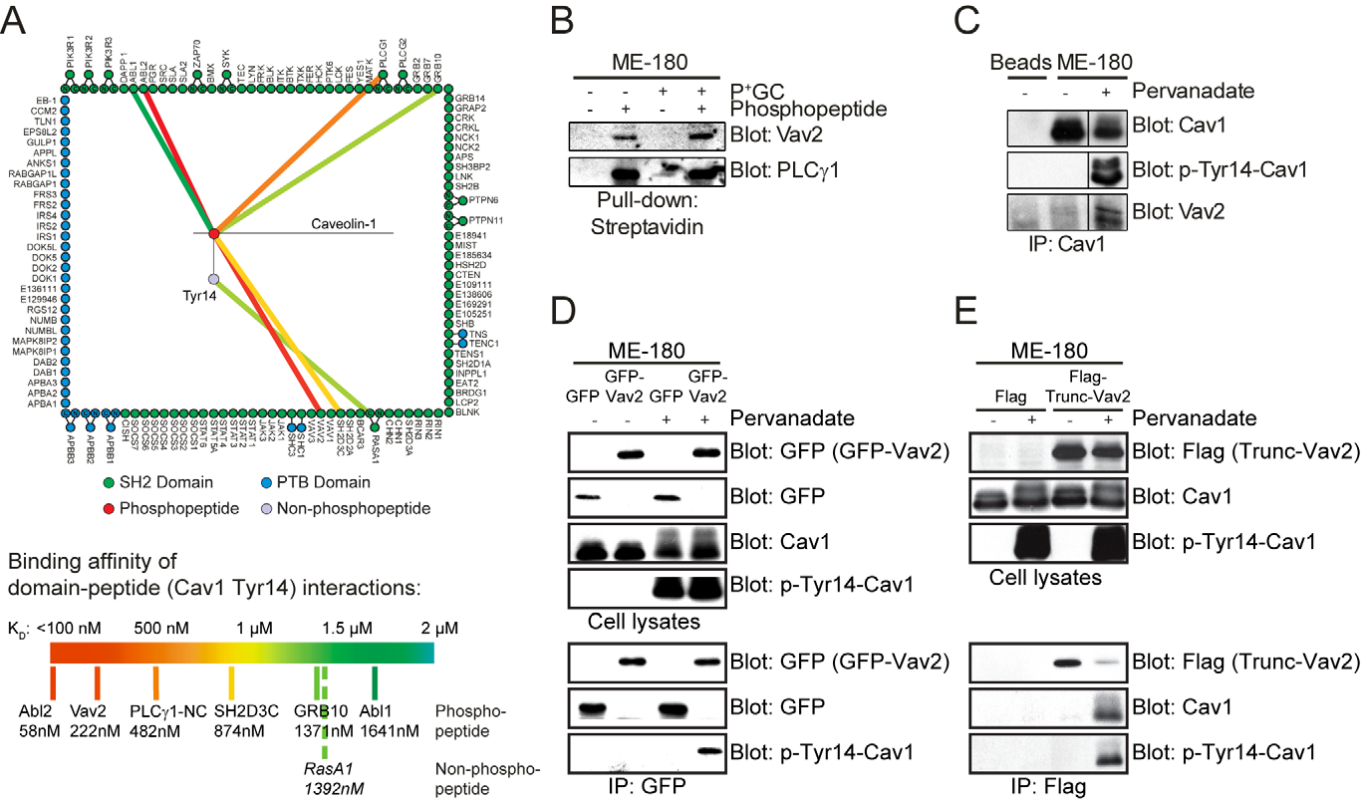
Phospho-Tyr14-Cav1 interacts strongly with the Rho-GEF Vav2. (A) A comprehensive, quantitative screen using microarrays of recombinant human SH2 and PTB domains reveals Tyr14-phosphorylation-dependent binding of Cav1 to several SH2-domain-containing proteins. The RhoA GEF Vav2 shows a high-affinity interaction (*K*_D_ of 222 nM) with the Tyr14-Cav1 phosphopeptide. The red circle represents the Tyr14-Cav1 phosphopeptide (18 amino acids); the purple circle represents the corresponding non-phosphorylated peptide; green and blue circles represent SH2 and PTB domains, respectively. Green or blue circles outside the rectangle represent tandem domains. The color of lines connecting peptides to domains indicates strength of observed interactions (see legend). *K*_D_ values for each hit are provided on the legend. (B) Vav2 and PLCγ1 co-precipitate with biotin-labeled Tyr14-Cav1 phosphopeptide. Western blot analysis of streptavidin precipitates of infected and uninfected ME-180 cells using biotin-labeled phosphorylated and non-phosphorylated Tyr14-Cav1 peptides as baits. Vav2 and PLCγ1 are found mainly in precipitates of the phosphorylated peptide. Levels of precipitated Vav2, but not PLCγ1, increase (by 40%) upon infection with P^+^GC. (C) Co-immunoprecipitation demonstrates increased protein-protein interaction of Cav1 and Vav2 after cell treatment with the phosphotyrosine phosphatase (PTP)-inhibitor pervanadate. Western blot analysis of immunoprecipitates of ME-180 cells using full-length Cav1 protein as bait. Cav1 was immunoprecipitated from untreated (−) and pervanadate-treated cells (+). Precipitates were probed for Cav1, phospho-Tyr14-Cav1, and Vav2. Tyrosine-phosphorylated Cav1 was precipitated exclusively from pervanadate-treated lysates. Levels of co-precipitated Vav2 are markedly increased in pervanadate-treated lysates. (D) GFP-Vav2 and Cav1 interact exclusively after cell treatment with pervanadate. Western blot analysis of total cell lysates (upper panels) and immunoprecipitates (lower panels) using heterologously expressed GFP-Vav2 (construct depicted in Figure S5, upper panel) or GFP protein as baits. Lysates were probed for GFP, Cav1, and phospho-Tyr14-Cav1, respectively. GFP-Vav2 and GFP were immunoprecipitated from untreated (−) and pervanadate-treated cells (+) using a GFP antibody. Precipitates were probed for GFP and phospho-Tyr14-Cav1. Phosphorylated Cav1 was recovered exclusively from pervanadate-treated lysates of GFP-Vav2 expressing cells. (E) Truncated Vav2 and Cav1 interact exclusively after cell treatment with pervanadate. Western blot analysis of ME-180 cell lysates (upper panels) and immunoprecipitates (lower panels) using heterologously expressed truncated Vav2 (construct depicted in Figure S5, lower panel) or FLAG-tag peptide as baits. Truncated Vav2 only possesses the C-terminal SH3-SH2-SH3 domains of Vav2. Using a FLAG antibody, truncated Vav2 was immunoprecipitated from untreated (−) and pervanadate-treated cells (+). Phosphorylated Cav1 was recovered exclusively from lysates of pervanadate-treated truncated Vav2 expressing cells. Data in A–E are representative of three independent experiments.

To identify signaling proteins that could interact with Cav1 upon phosphorylation at Tyr14, we synthesized two fluorescently labeled peptides with sequences corresponding to residues 5-22 of Cav1, one phosphorylated on Tyr14 and the other not phosphorylated. We then used these peptides to probe protein microarrays comprising virtually every Src homology 2 (SH2) and phosphotyrosine binding (PTB) domain encoded in the human genome, as previously described [28]. In order to obtain quantitative information, we probed the arrays, in duplicate, with eight concentrations of each peptide, ranging from 10 nM to 5 µM. We then fit the resulting fluorescence data to an equation that describes saturation binding [28], enabling us to obtain equilibrium dissociation constants (*K*_D_s) for the binding of each peptide to each recombinant domain (Figure 3A). Previous studies with other phosphopeptides have shown that >90% of the SH2 and PTB domains on these arrays are active [28] and hence non-interactions should be viewed as reliable information as well.

In total, the arrays highlighted six SH2 domains that recognized the Cav1 phosphopeptide with high affinity (*K*_D_ <2 µM): Abl2, Vav2, Phospholipase Cγ1 (PLCγ1), SH2D3C, Grb10, and Abl1. Although strong interactions with the SH2 domain of Abl2 are frequently observed (this is a particularly promiscuous domain), we were intrigued by the high affinity interaction with the SH2 domain of RhoA GEF Vav2 (*K*_D_ = 220 nM). To investigate the physiological relevance of this biophysical interaction, we performed the following biochemical experiments: First, we incubated biotin-labeled peptides with sequences corresponding to residues 7–21 of Cav1, one phosphorylated on Tyr14 and the other not phosphorylated, with cellular lysates derived from ME-180 cells and precipitated the peptides with streptavidin-coated beads. Consistent with the microarray data, Vav2 co-purified exclusively with the phosphorylated peptide (Figure 3B). Moreover, 40% more Vav2 was recovered from infected cell lysates than from uninfected cells. Similarly, PLCγ1, another important binding partner identified by the protein microarray, showed a vastly increased binding affinity to the phosphorylated peptide. However, levels of PLCγ1 were similar in both lysate types. Next, we immunoprecipitated full-length, endogenous Cav1 protein from either untreated ME-180 cells or from cells that had been pretreated with the tyrosine phosphatase inhibitor pervanadate to trigger elevated levels of Cav1 phosphorylation. Western blot analysis revealed binding between Vav2 and full-length Cav1 in pervanadate-treated cells (Figure 3C). To better understand the molecular mechanism of the observed phospho-Tyr14-Cav1–Vav2 interaction we expressed different Vav2 constructs (Figure S5) in ME-180 cells and immunoprecipitated them using antibodies against the respective tags [29],[30]. First, we expressed the full-length Vav2 coupled with GFP in pervanadate- and control-treated cells. We were then able to precipitate GFP-Vav2 and control GFP from transfected cells using a GFP antibody; however, phospho-Tyr14-Cav1 co-precipitated exclusively with pervanadate-treated GFP-Vav2 expressing cells (Figure 3D). This further demonstrated the phospho-specificity of the phospho-Tyr14-Cav1–Vav2 protein-protein interaction.

Since only the SH2 domain of the Vav2 protein had been spotted on the protein microarray, we assumed the Vav2-Cav1 interaction was SH2-specific. To verify this, we expressed truncated Vav2 coupled to FLAG in pervanadate- and control-treated cells. Truncated Vav2 consists solely of the C-terminal SH3-SH2-SH3 domains of the protein (Figure S5). Similar to full-length Vav2, we were able to precipitate truncated Vav2 from transfected cells using a FLAG antibody. Again, phospho-Tyr14-Cav1 co-precipitated exclusively with pervanadate-treated, truncated Vav2 expressing cells (Figure 3E), demonstrating the relevance of the remaining Vav2 domains for the observed phospho-Tyr14-Cav1–Vav2 interaction. Taken together, these results identify the RhoA GEF Vav2 as a novel interaction partner of tyrosine-phosphorylated Cav1.

**Figure 4:**
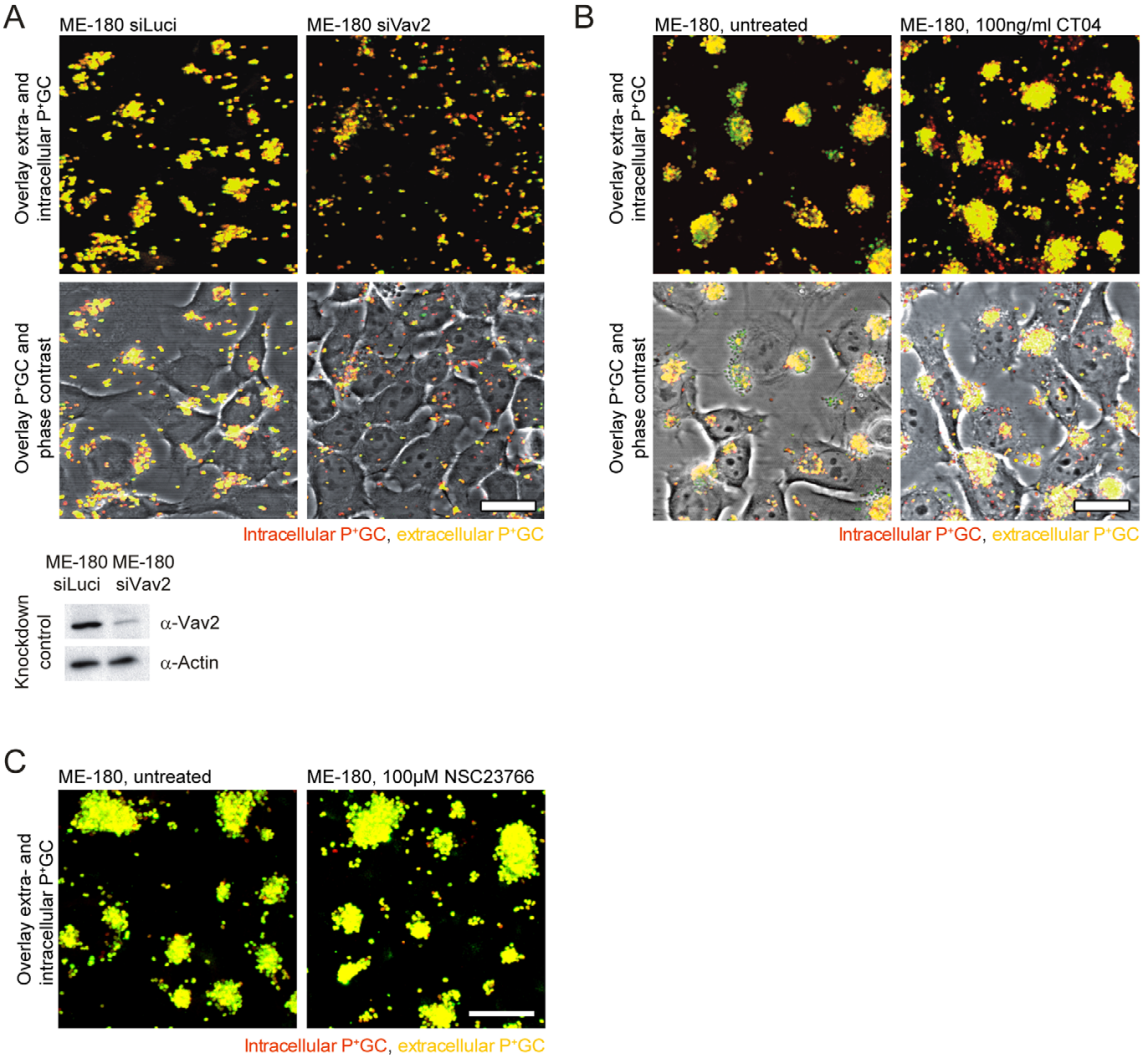
Vav2 and RhoA prevent internalization of P^**+**^GC by host cells. (A) Knockdown of Vav2 levels in ME-180 cells using siRNA results in P^+^GC internalization. Intracellular bacteria (red) are detected in siVav2-treated ME-180 cells (upper right panels), whereas only extracellular bacteria (yellow-green) are detected in siLuciferase-treated control cells (upper left panels). Efficiency of Vav2 knockdown in ME-180 cells after siRNA treatment (lower panel). Scale bar: 20 µm. (B) Treatment of ME-180 cells with the membrane-permeable Rho inhibitor CT04 results in P^+^GC internalization. Intracellular bacteria (red) are detected in CT04-treated ME-180 cells (right panels), whereas only extracellular bacteria (yellow-green) are detected in control-treated ME-180 cells (left panels). Scale bar: 20 µm. (C) Treatment of ME-180 cells with the Rac1 inhibitor NSC23766 does not result in P^+^GC internalization. Only extracellular bacteria (yellow-green) are detected in 100 µM NSC23766-treated ME-180 cells (right panel) and control-treated ME-180 cells (left panel). Scale bar: 20 µm. Data in A–C are representative of three independent experiments.

Next, we assessed the role of Vav2 and PLCγ1 in P^+^GC infection by knocking down their function in ME-180 cells using RNAi. Confocal microscopy revealed that, as with Cav1, reducing Vav2 levels using siRNA resulted in efficient internalization of P^+^GC by ME-180 cells (Figure 4A). By contrast, shRNA-mediated downregulation of PLCγ1 in ME-180 cells did not result in P^+^GC internalization (Figure S6A). This shows that Vav2 plays a role in preventing bacterial uptake, possibly by participating in cytoskeletal reorganization through its function as a GEF for the Rho/Rac family of GTPases. Alternatively, Vav2 could function in a manner independent of its GEF activity simply by physically linking signaling molecules to the actin cytoskeleton [31]. To test for the involvement of RhoA in impeding bacterial internalization, we treated ME-180 cells with low concentrations of the Rho-specific inhibitor CT04, a cell permeable form of the C3 transferase from *Clostridium botulinum*, and subsequently infected the cells with P^+^GC. Interestingly, treatment with CT04 led to a strong uptake of bacteria (Figure 4B). Since Vav2 also activates Rac1 [32], we also tested the relevance of Rac1 for P^+^GC internalization. ME-180 cells were treated with the Rac1-specific chemical inhibitor NSC23766 [33] and then infected with P^+^GC. In contrast to Rho inhibition, treatment with NSC23766 did not affect bacterial entry (Figure 4C). These results were also confirmed in ME-180 and HeLa epithelial cells using different inhibitor concentrations (Table S1). Partial siRNA-mediated knockdown of RhoA further demonstrated the relevance of this small GTPase in impeding cellular uptake of P^+^GC. Interestingly, downregulation of other small GTPases such as Cdc42 and Rac1 did not enhance P^+^GC internalization (Figure S6B and Table S2). Together, these findings highlight the importance of RhoA in preventing bacterial entry, probably by forming a cytoskeletal barrier.

**Figure 5:**
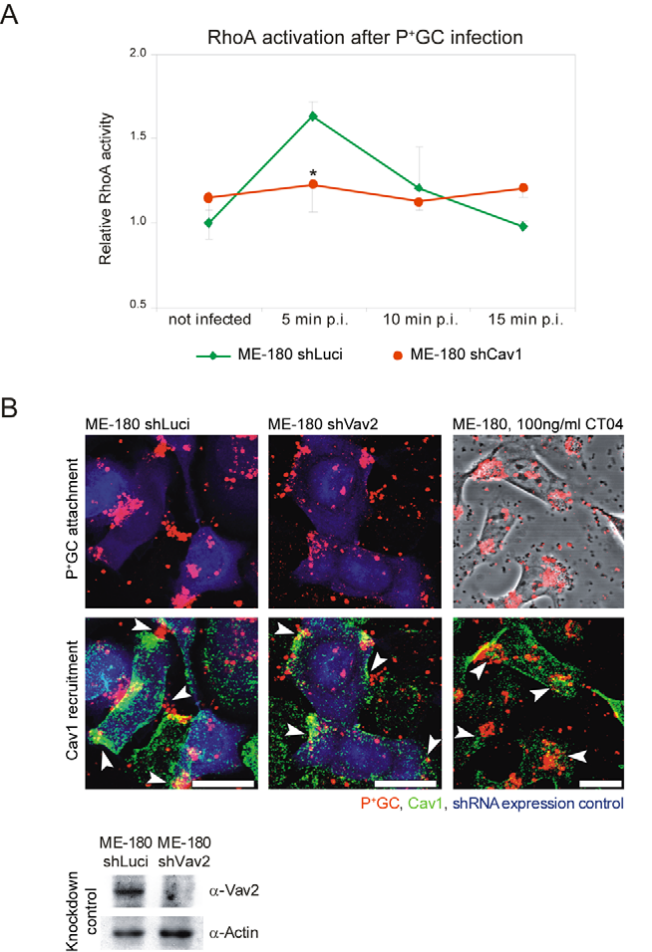
Vav2 and RhoA act as downstream signaling partners of Cav1 during P^**+**^GC infection. (A) RhoA activation after P^+^GC infection depends on Cav1 expression. Levels of the active, GTP-bound state of RhoA are compared between ME-180 shCav1 knockdown cells and ME-180 shLuciferase control cells during an infection time-course. Data are mean ± standard deviation of triplicate wells after normalizing protein levels. RhoA activity was normalized to uninfected ME-180 shLuciferase cells. (B) Cav1 recruitment does not depend on Vav2 expression or RhoA activation. Cav1 (green) recruitment to attached P^+^GC (red) is observed in ME-180 shLuciferase control cells (blue, upper left panels), ME-180 shVav2 knockdown cells (blue, upper middle panels), and Rho inhibitor CT04-treated ME-180 cells (upper right panels). Recruited Cav1 is indicated by arrows. Efficiency of Vav2 knockdown in ME-180 cells after lentiviral transduction of luciferase (control) or Vav2 shRNA constructs (lower panel). Scale bar: 20 µm. Images are representative of three independent experiments.

To investigate the direct impact of Cav1 expression on RhoA activation, we compared RhoA activation in control ME-180 cells expressing a luciferase shRNA with Cav1 shRNA knockdown cells in response to infection with P^+^GC. Control cells showed a strong increase in the levels of the GTP-bound state of RhoA within the first 5 min of infection, followed by a decline to basal levels over the next 10 min. By contrast, Cav1 knockdown cells did not activate RhoA throughout the early stages of infection (*p*<0.05, Figure 5A). These data support a model in which a signaling cascade involving Cav1, Vav2, and RhoA act to inhibit the internalization of P^+^GC by host cells. Finally, to delineate the sequence of the Cav1-Vav2-RhoA signaling cascade, we infected both Vav2-knockdown ME-180 cells and CT04-treated ME-180 cells with P^+^GC and monitored the recruitment of Cav1 by confocal microscopy (Figure 5B). Cav1 recruitment was not affected by either treatment, indicating that Cav1 lies upstream of Vav2 and RhoA. Taken together, our data strongly suggest that P^+^GC infection induces a Cav1-Vav2-RhoA signaling cascade in host cells. Phosphotyrosine-dependent Cav1 recruitment to sites of bacterial attachment induces the recruitment of Vav2 and RhoA in response to infection, which functions to prevent bacterial internalization, probably via RhoA-dependent cytoskeletal rearrangements.

Our findings highlight a compelling anti-invasive strategy of pathogenic bacteria, uncover Vav2 as a novel Cav1 signaling partner, and suggest ways in which tyrosine-phosphorylated-Cav1 could mediate cytoskeletal rearrangements. In addition to identifying Vav2 as a Cav1 interaction partner, our protein microarrays highlighted five other proteins, all of which have been implicated in modulating the cytoskeleton through small GTPases: SH2D3C is an integrin-associated signaling pathway component, proposed to regulate the actin cytoskeleton via its GEF-like domain which binds Ras family GTPases [34]; PLCγ1 exhibits mitogenic activity by acting as a GEF for the small GTPase PIKE (phosphatidylinositol-3-OH kinase (PI(3)K) enhancer) [35] and is also capable of directly activating Rac1 [36]; Grb10 putatively binds small GTPases of the Ras superfamily [37]; and the Abl kinases are known to link various cell surface receptors to signaling pathways involved in cytoskeletal reorganization and to regulate the activation of Rac and Rho GTPases [38],[39]. It remains to be determined if these additional proteins play a role in Cav1-mediated signaling.

Recently, Cav1 has been demonstrated to interact with RhoA and Rho-associated kinase 1 (ROCK1) to promote Rho activation through inhibition of the Src-p190RhoGAP pathway. Cav1 has also been shown to serve as a target of ROCK1 signaling, indicating the presence of a positive feedback loop [40]–[42]. Furthermore, phospho-Tyr14-Cav1 orders microdomains within focal adhesions and stabilizes focal adhesion-associated kinase [43],[44]. Here, we report a direct link between RhoA activation and tyrosine-phosphorylated Cav1 through the RhoA GEF Vav2. Phosphorylation of Cav1 on Tyr14 may serve as a crucial switch between the activated and inactivated state of RhoA. Thus, the direct interaction of tyrosine-phosphorylated Cav1 with Vav2 might support increased activation of RhoA in cellular compartments where tyrosine-phosphorylated Cav1 accumulates, such as focal adhesions or at sites of P^+^GC attachment.

Together, our data reveal an immediate early anti-invasive activity of P^+^GC, dependent on tyrosine-phosphorylated Cav1 host-cell signaling, that facilitates the establishment and maintenance of this pathogen's extracellular niche. This process, triggered by Vav2-mediated activation of RhoA, elicits cytoskeletal rearrangements that may function as a physical barrier to prevent internalization of attached bacteria. Cav1 is known to participate in protein trafficking [18], and we have observed a drastic, instantaneous, and RhoA-independent recruitment of Cav1 at sites of P^+^GC attachment. Thus, Cav1 is the presumptive central element of the identified Cav1-Vav2-RhoA signaling cascade, playing a crucial role in the localization of its signaling partners to the site of infection. The resulting prevention of premature uptake seems to be beneficial for these bacteria as P^+^GC are rapidly killed inside host cells. P^+^GC, and probably other Tfp-producing bacteria including EPEC, could use this initial extracellular phase to adapt and prepare for the subsequent steps of infection. For example, GC employ variation of the Opa invasins to prepare individual bacteria for deliberate cell entry [10] and transcytosis [45]. EPEC, on the other hand, may use their Tfp as an immediate block to cell entry even before the TTSS is placed or used in delivering its anti-invasive effectors [46]. Taken together, this Tfp-triggered mechanism extends our understanding of how P^+^GC use pili to elicit cytoprotective effects and modulate the host cell for their own benefit, as described previously [5],[6]. By investigating P^+^GC colonizing its host, we have exploited these bacteria as a tool to identify an anti-invasive bacterial strategy as well as a novel Cav1-dependent signaling cascade leading to RhoA activation.

## Methods

### Cell Lines and Bacterial Infection

The human cervix carcinoma cell line ME-180 (ATCC HTB33) was grown in McCoy's 5A medium (Gibco-Invitrogen, Carlsbad, CA, USA) supplemented with 10% FCS (Biochrom, Berlin, Germany). The human gastric adenocarcinoma cell line AGS (ATCC CRL-1739) was grown in RPMI 1640 medium (Gibco-Invitrogen, Carlsbad, CA, USA) supplemented with 10% FCS. For microscopy, cells were seeded on acid-washed glass coverslips. The GC strains used in this work were derived from *N. gonorrhoeae* strain MS11 [47]. Strain P^+^GC was selected for observable piliated phenotype (P^+^Opa^−^). P^+^GC, P^+^GCΔ*pilT* [48] and non-piliated Opa_57_-expressing GC [49] strains were resuspended in cell culture medium and added to cell monolayers in serum-free medium at a multiplicity of infection of 100. EPEC strains E2348/69 (O127:H6) and EPEC 2348/69 CVD452, a mutant defective in type III-dependent secretion, were grown overnight at 37°C in LB broth without shaking. The following day cultures were diluted 1∶100 in serum free DMEM and grown without shaking under previously described conditions known to stimulate TTSS expression for 3.5 h to create so-called preactivated cultures [50]. Consequently EPEC cultures were added to cell monolayers for 2 h. Cells were treated with either 100 µM pervanadate (Sigma Aldrich, St. Louis, MO, USA), 1 µM Cyt D (Sigma Aldrich, St. Louis, MO, USA) or 100 nM Lat A (Biomol, Hamburg, Germany) for 30 min, 10 µM PP2 (Calbiochem, San Diego, CA, USA), 10 µM STI571 (LC Labs, Woburn, MA, USA), or 20 µM, 100 µM, and 300 µM NSC23766 (Calbiochem, San Diego, CA, USA) for 1 h or 50 ng/ml, 100 ng/ml, and 250 ng/ml cell permeable Rho inhibitor CT04 (Cytoskeleton, Denver, CO, USA) for 4 h before infection. Experimental treatments had no effect on bacterial viability.

### Gentamicin Protection Assay

Quantification of bacterial binding and entry into host cells was performed using standard gentamicin-based assays with dilution plating to recover viable bacteria. Cell confluency at infection time was 70%. Cells were washed three times in serum-free RPMI 1640 medium prior to infection and incubated in serum-free RPMI 1640 medium (with indicated chemicals) for 30 min. Bacteria were added to the cells at a multiplicity of infection of 100. Cells were then incubated in RPMI 1640 medium at 37°C, 5% CO_2_ for 2 h. 100 µg/ml gentamicin (Sigma Aldrich, St. Louis, MO, USA) was then added for an additional 2 h to kill extracellular bacteria. Cells were washed, and 1% saponin (Serva, Heidelberg, Germany) was added to permeabilize cells followed by plating of appropriate dilutions of the lysate on GC agar. To quantify adherent bacteria, lysis with saponin was done prior to gentamicin treatment. Intracellular gentamicin-protected (Gm^P^) bacteria were determined as a percentage of total cell-associated bacteria. Assays were conducted in triplicate wells, yielding the given mean and the standard deviation. Each experiment was repeated at least three times. Data were tested for significance using Student's *t* test**.** Representative experiments are shown.

### Cloning, Plasmids, and Transfection

The coding region of human *caveolin-1* (*cav1*) was amplified from total cDNA of the ME-180 cell line and cloned into the expression vector pcDNA3 (Promega, Madison, WI, USA), which also encodes an N-terminal HA-tag. For live-cell microscopy, *cav1* was cloned into the vector pEGFP-N1 (Invitrogen, Grand Island, NY, USA). AGS cells were transfected with the vector pcDNA3 alone or the pcDNA3-*cav1* construct (Cav1-HA), then stable clones were isolated and maintained in RPMI supplemented with 10% FCS and 500 µg/ml G418 (PAA Laboratories, Linz, Austria). The point mutation Y14F-Cav1-HA was generated by changing tyrosine 14 of *cav1* in Cav1-HA to phenylalanine using the QuikChange site-directed mutagenesis kit (Stratagene, La Jolla, CA, USA) according to the manufacturer's protocol. The accuracy of the mutation was confirmed by DNA sequencing. Full-length GFP-Vav2 cloned into pEGFP-C2 was a gift of Dr. László Buday (Semmelweis University, Budapest, Hungary) and truncated Vav2 (consisting of the C-terminal SH3-SH2-SH3 domains of Vav2) cloned into pcDNA3. FLAG was a gift of Dr. Daniel D. Billadeau (Mayo Clinic, Rochester, MN, USA). All transfections were performed using Lipofectamin™ 2000 (Invitrogen, Grand Island, NY, USA) according to the manufacturer's protocol. The cells were further analyzed 24 h post-transfection.

### RNAi

The siRNA duplexes targeting human Cav1 (Cav1A: GCAGTTGTACCATGCATTA, Cav1B: ATTAAGAGCTTCCTGATTG), RhoA (TAGGCTGTAACTACTTTATAA), Rac1 (ATGCATTTCCTGGAGAATATA), Cdc42 (TTCAGCAATGCAGACAATTAA), and firefly luciferase (AACUUACGCUGAGUACUUCGA) were purchased from Qiagen (Hilden, Germany). The siRNA duplexes targeting lamin A (CCTGGACTTCCAGAAGAACA) and Vav2 (ON-TARGET Plus SMART pool containing the following siRNAs: CUGAAAGUCUGCCACGAUA, UGGCAGCUGUCUUCAUUAA, GUGGGAGGGUCGUCUGGUA, and GCCGCUGGCUCAUCGAUUG) were synthesized by Dharmacon Research (Lafayette, CO, USA). The transfection of siRNAs was carried out using Hiperfect transfection reagent (Qiagen, Hilden, Germany) according to the manufacturer's instructions. Briefly, ME-180 and AGS-Cav1 cells were transfected with 50 nM siRNA duplex and used for experiments 72 h after transfection.

### Generation of shRNA Knockdown Cell Lines

shRNA-expressing vectors were constructed by cloning computed shRNA oligonucleotides (Metabion, Martinsried, Germany) into the pLVTHM vector. The sequences of the targets of the shRNAs are as follows: human Cav1, 5′-CAGCAACAATTTATGAATTGA-3′; human Vav2, 5′-GCATGACTGAAGATGACAAGA-3′; human PLCγ1, 5′-GGACTTTGATCGCTATCAAGA-3′; firefly luciferase, 5′-AACTTACGCTGAGTACTTCGA-3′. All constructs were verified by sequencing. Viruses carrying the shRNAs were produced by transfecting 293T cells with the generated pLVTHM constructs together with viral packaging vectors (psPAX2, pMD2G, kindly provided by D. Trono, Ecole Polytechnique Fédérale de Lausanne, Switzerland) by calcium phosphate transfection. Viruses were harvested from the supernatant 48 h after transfection, filtered, and applied to ME-180 cells for lentiviral infection in the presence of polybrene (5 µg/ml, Sigma-Aldrich, St. Louis, MO, USA). Pools of GFP-positive cells were selected and validated for their ability to knock down protein expression of target genes by more than 70% in comparison with luciferase control cells.

### Microscopy

Cells were grown on coverslips (12 mm diameter) and processed for immunofluorescence as described previously [51]. Differential staining of intra- and extracellular bacteria was achieved by double staining of bacteria, primarily without permeabilization of cells and subsequently after cell permeabilization. For labeling, the following antibodies were used: anti-Cav1 (N20, Santa Cruz Biotechnology, Santa Cruz, CA, USA), rabbit anti-*Neisseria gonorrhoeae* (USBiological, Swampscott, MA, USA), and anti-pilus (m346, monoclonal mouse, generated at the Max Planck Institute for Infection Biology). All antibodies were used at a 1∶100 dilution. Filamentous actin was detected with Alexa 546-conjugated phalloidin (Invitrogen, Grand Island, NY, USA). All secondary antibodies were purchased from Jackson Immuno Research Laboratories (West Grove, PA, USA). Samples were analyzed by confocal laser scanning microscopy using a Leica TCS SP microscope, equipped with an argon/krypton mixed gas laser source (Leica, Solms, Germany). Image stacks were further processed using Photoshop (Adobe Systems, San Jose, CA, USA) or Imaris (Bitplane, Zürich, Switzerland).

### Live-Cell Confocal Microscopy

ME-180 cells were transfected with the described pEGFP-N1-Cav1 construct and grown in 3.5 cm^2^ glass-bottom dishes (MatTek, Ashland, MA, USA) overnight under standard conditions. Fresh serum-free RPMI without phenol red (Gibco-Invitrogen, Carlsbad, CA, USA) was added, and cells were placed in a humidified incubation chamber at 37°C and 5% CO_2_. Images were obtained with the VT-Infinity system (Visitron Systems, Munich, Germany). Briefly, the system consists of an Olympus IX81 (Olympus, Tokyo, Japan), VT-Infinity galvo scanner confocal head (Visitron Systems, Munich, Germany), and a Hamamatsu C9100-02 CCD camera (Hamamatsu Photonics K.K, Tokyo, Japan). Bright field images were acquired with a 63× phase contrast objective (NA1.25 oil, Olympus, Tokyo, Japan) and a high-speed shutter system. Fluorescent images were acquired with a 488 nm laser beam with an intensity of 250 mW using the 488 nm emission filter set (Chroma Technology, Brattleboro, VT, USA). Images were collected and processed using MetaMorph (Universal Imaging Corporation, West Chester, PA, USA) and Imaris (Bitplane, Zürich, Switzerland) software.

### Immunoprecipitations and Immunoblots

Pervanadate-treated and untreated ME-180 cells were lysed in 1× Cell Lysis Buffer (Cell Signaling, Boston, MA, USA) containing PhosStop Phosphatase Inhibitor and Complete™ Protease Inhibitor (both: Roche Diagnostics, Mannheim, Germany). The lysates were pre-cleared for 4 h with protein G-agarose beads (Calbiochem, San Diego, CA, USA) and incubated with 2 µg anti-Cav1 antibody (rabbit, BD Transduction Laboratories, Franklin Lakes, NJ, USA) overnight. Protein G-agarose beads were subsequently added for 4 h to precipitate antigen-antibody complexes. After extensive washing, the precipitate was eluted by heating to 95°C in SDS loading buffer and the individual proteins separated by SDS-PAGE. Western blotting was used to assess the precipitate using the following antibodies: anti-Cav1 (N20, Santa Cruz Biotechnology, Santa Cruz, CA, USA), anti-phospho-Tyr14-Cav1 (clone 56, BD Transduction Laboratories, Franklin Lakes, NJ, USA) and anti-Vav2 (C64H2, Cell Signaling, Boston, MA, USA), and HRP-conjugated secondary antibodies (Amersham Biosciences, Pittsburgh, PA, USA). Western blot was developed using ECL reagent (ICN Biomedicals, Aurora, OH, USA). Blots were quantified using ImageJ software (v1.44a).

### Peptide Synthesis and Protein Arrays

Fluorescently labeled peptides with sequences corresponding to residues 5–22 of Cav1 were synthesized, one phosphorylated on Tyr14 and the other not phosphorylated as previously described [28], purified to >95% by preparative reverse phase HPLC, and quality controlled via mass spectrometry and analytical HPLC (Thermo Electron, Karlsruhe, Germany). Human SH2 and PTB domains were expressed and purified as previously described [28], and protein microarrays were fabricated and probed as more recently reported [52].

### Peptide Synthesis and Streptavidin-Agarose Pull-Down

Peptides were designed as 15-mers (residues 7–21 of Cav1) bearing an N-terminal biotin. Peptides were synthesized as pairs, one phosphorylated on Tyr14 and the other not phosphorylated, purified to >95% by preparative reverse phase HPLC, and quality controlled via mass spectrometry and analytical HPLC (Thermo Electron, Karlsruhe, Germany). For affinity pull-downs, 10 nmol of immobilized peptide was added to ∼2 mg of cell lysate. ME-180 cells were lysed in 1× Cell Lysis Buffer (Cell Signaling, Boston, MA, USA) containing 2 mM sodium orthovanadate, as a phosphatase inhibitor, and Complete™ Protease Inhibitor (Roche Diagnostics, Mannheim, Germany). The lysates were pre-cleared for 1 h with streptavidin agarose beads (Invitrogen, Grand Island, NY, USA) and equal amounts of lysate were incubated overnight at 4°C with streptavidin agarose beads, pre-saturated with the respective biotinylated peptides. After extensive washing, the streptavidin precipitate was eluted by heating to 95°C in SDS loading buffer and the individual proteins separated by SDS-PAGE. Western blotting was used to assess the precipitate using anti-Vav2 (C64H2, Cell Signaling, Boston, MA, USA) antibody.

### Cytoskeletal Preparation

AGS and ME-180 cells (2×10^7^) were washed with PBS at 4°C and then incubated in lysis buffer (1 mM EGTA, 4% PEG 6000, 100 mM PIPES pH 6.9, 0.5% Triton X-100) for 5 min at 4°C to stabilize the cytoskeleton. Supernatant containing cytoplasmic and compartmental proteins were removed and the remaining cytoskeletal proteins washed once, harvested in lysis buffer by scraping, and pelleted by centrifugation (14,000×g, 5 min). Pellets were washed once with 1 ml wash buffer (1 mM EGTA, 4% PEG 6000, 100 mM PIPES pH 6.9), then collected in SDS loading buffer and analyzed by Western blotting.

### RhoA Activation Assay

Using an enzyme-linked immunosorbent assay-based RhoA activation assay kit (Cytoskeleton, Denver, CO, USA) active RhoA was determined according to the manufacturer's protocol. Briefly, to synchronize Rho activity, cell monolayers exhibiting 60% confluency were grown in culture medium with 0.5% FCS for an additional 24 h and then serum-starved for another 16 h. After infection, cells were lysed at the indicated time points, aliquots snap-frozen in liquid nitrogen, and the protein concentration determined using Precision Red Advanced Protein Assay (Cytoskeleton, Denver, CO, USA). Cell lysate (37.5 µg protein) from each sample was incubated in microwells coated with the isolated Rhotekin Rho-binding domain. Active RhoA was subsequently measured using immunodetection followed by a colorimetric reaction measured by absorbance at 490 nm. Assays were conducted in triplicate microwells, yielding the given mean and the standard deviation. Data were tested for significance using Student's *t* test**.**

### Immunogold Labeling

Cells were fixed in 2% PFA/1% acrolein in PBS for 2 h at RT. After washing with PBS, the cells were overlaid with warm gelatine (10% PBS) and scraped off the plate. After gelling at 4°C, the specimens were cut into small blocks, post-fixed in 2% PFA, and infiltrated with a sucrose/PVP solution. Specimens were mounted on a stub, frozen in liquid nitrogen, and 60 nm sections were produced using a RMC MTX/CRX cryo-ultramicrotome (Boeckeler Instruments, Tucson, AZ, USA). Sections were thawed, blocked, and incubated with anti-Cav1 antibody (rabbit, BD Transduction Laboratories, Franklin Lakes, NJ, USA). After washing, bound antibody was detected using anti-rabbit secondary antibodies coupled to 6 nm colloidal gold. The samples were analyzed on a Leo 906E transmission electron microscope (Carl Zeiss, Jena, Germany) equipped with a Morada digital camera (Silicon Integrated Systems, Hsinchu, Taiwan).

### Generation of Anti-Pilus Antibody

P^+^GC pili were purified as described previously [53]. Purified pili were utilized for immunizing BALB/c mice for the generation of monoclonal antibodies following standard poly-ethylene glycol (PEG) fusion protocol. Briefly, 6–8-wk-old Balb/c mice were primed with 50 µg of purified pili in Freund complete adjuvant followed by two boost injections on day 20 and 40 in Freund incomplete adjuvant. Spleen cells were fused with P3X63Ag8 myeloma cells. Positive hybridomas were screened by standard ELISA against purified pili. Anti-pilin antibody producing hybridomas were subcloned three times by limited dilution.

## Supporting Information

Figure S1 **The inhibitory effect of Cav1 on P^**+**^GC internalization is pili-specific and independent of pili retraction.** (A) Attachment of non-piliated P^−^Opa_57_^+^GC does not induce Cav1 (white, middle panel) recruitment 2 h post-infection. (B) Expression of Cav1 in AGS cells does not inhibit P^−^Opa_57_^+^GC internalization. Mock-transfected AGS cells and AGS-Cav1 cells were infected with P^+^GC or P^−^Opa_57_^+^GC. (C) Cav1 inhibits uptake of the P^+^GCΔ*pilT* mutant. AGS-Mock and AGS-Cav1 cells were infected with P^+^GC or the P^+^GCΔ*pilT* mutant. Gentamicin protection assays were performed 2 h post-infection. Intracellular gentamicin protected (Gm^P^) bacteria were determined as a percentage of total cell-associated bacteria, which were comparable for the different host cells. Experiments were performed in triplicate. Error bars indicate mean ± standard deviation.
(0.36 MB TIF)

Figure S2 **Tfp-producing EPEC induce Cav1 accumulation and prevent host cell entry.** (A) ME-180 cells were infected with pre-activated cultures of wild-type EPEC strain E2348/69 and EPEC 2348/69 CVD452, a type III secretion system (TTSS) defective mutant, for 2 h. Endogenous Cav1 (green) is recruited to attachment sites of the microcolony-forming mutant, whereas the TTSS-preactivated wild type adheres dispersed and does not trigger Cav1 recruitment. (B) AGS and AGS-Cav1 cells were infected with E2248/69 wild type and TTSS CVD452 mutant EPEC. Intracellular gentamicin protected (Gm^P^) bacteria were determined as a percentage of total cell-associated bacteria. Experiments were performed in triplicate. Error bars indicate mean ± standard deviation.
(0.89 MB TIF)

Figure S3 **Immunogold labeling of Cav1 in ME-180 cells after infection with P^**+**^GC.** Cav1 is 6-nm-gold-labeled (black arrows). P^**+**^GC are observed as diplococci attached to the cell membrane (white arrows). Scale bar: 500 nm.
(3.63 MB TIF)

Figure S4 **Depolymerization of F-actin induces bacterial internalization and impedes Cav1 recruitment.** (A) Disruption of F-actin filaments (red) with cytochalasin D (CytD) prevents Cav1 (white, lower panel) recruitment to P^+^GC attachment sites (green). Cellular borders are represented as yellow outlines. Scale bars: 20 µm. (B) ME-180 cells were treated with Cyt D or latrunculin A (Lat A), which disrupt actin filaments. Gentamicin protection assay was performed 2 h post-infection. Intracellular Gm^P^ bacteria were determined as a percentage of total cell-associated bacteria. Ratio of Gm^P^ P^+^GC calculated relative to untreated control. Experiments were performed in triplicate. Error bars indicate mean ± standard deviation.
(1.09 MB TIF)

Figure S5 **(A) shRNA-mediated downregulation of PLCγ1 in ME-180 cells does not result in P^**+**^GC internalization.** Only extracellular bacteria (yellow-green) are detected in ME-180 shPLCγ1 cells (upper right panels) and ME-180 shLuciferase control cells (upper left panels). Efficiency of PLCγ1 knockdown in ME-180 cells after lentiviral transduction of luciferase (control) or PLCγ1 shRNA constructs (lower panel). Scale bar: 20 µm. (B) Knockdown of RhoA but not Rac1 or Cdc42 in ME-180 results in P^+^GC internalization. Intracellular bacteria (red) are detected in siRhoA-treated cells (upper panel, lower right image), whereas only extracellular bacteria (yellow-green) are detected in siCdc42-treated (upper right image), siRac1-treated (lower left image), and siMock-treated cells (upper left image). Knockdown efficiencies of Cdc42, Rac1, and RhoA after siRNA treatment (lower panel; see also Table S2). Scale bar: 20 µm. Data are representative of three independent experiments.
(1.15 MB TIF)

Figure S6 **Vav2 constructs used in this work. Full-length GFP-Vav2 cloned into pEGFP-C2 (upper panel) and truncated Vav2 cloned into pcDNA3.FLAG (lower panel).** Truncated Vav2 only possesses the C-terminal SH3-SH2-SH3 domains of Vav2.
(0.17 MB TIF)

Table S1 **Synopsis of Rho- and Rac1-inhibitor experiments shows the importance of Rho to prevent P^**+**^GC uptake in different cell lines.** The results of eight experiments are summarized here. CT04 exhibited dose-dependent uptake effects in ME-180 as well as HeLa cells in all experiments, whereas, in general, NSC23766 did not impact P^+^GC internalization. To determine uptake rate—i.e., mild, (+); effective, +; strong, +++—25 or more image stacks of treated and untreated cells per experiment were analyzed for bacterial uptake and cell survival.
(0.16 MB TIF)

Table S2 **Synopsis of siRNA-mediated knockdown experiments of small GTPases Cdc42, Rac1, RhoA underscores the importance of RhoA for preventing P^**+**^GC uptake.** Results of five experiments are summarized here. In three out of four experiments knockdown of RhoA in ME-180 cells led to increased uptake of P^+^GC. By contrast, internalized bacteria were detected in only one out of four experiments after siRNA mediated downregulation of Cdc42 and Rac1. To determine uptake rate—i.e., mild, (+); effective, +; strong, +++—25 or more image stacks of treated and untreated cells per experiment were analyzed for bacterial uptake and cell survival.
(0.18 MB TIF)

Video S1 **Rapid recruitment of Cav1 to sites of P^**+**^GC adherence.** Cav1-GFP (green), time frame 0 to 92 min.
(10.36 MB WMV)

## Abbreviations

Cav1: caveolin-1
Cyt D: cytochalasin D
EPEC: enteropathogenic *Escherichia coli*
GEF: guanine nucleotide exchange factor
Lat A: latrunculin A
MC: microcolony
Opa: opacity associated proteins
P^+^GC: piliated *Neisseria gonorrhoeae*
PIKE: phosphatidylinositol-3-OH kinase (PI(3)K) enhancer
PLCγ1: Phospholipase Cγ1
PTB: phosphotyrosine binding
PTP: phosphotyrosine phosphatise
RNAi: RNA interference
ROCK1: Rho-associated kinase 1
SH2: Src homology 2
TfP: type IV pili
TTSS: type III secretion system
Tyr14: tyrosine 14
