# Supplementary figures and images for: Tyrosine-Phosphorylated Caveolin-1 Blocks Bacterial Uptake by Inducing Vav2-RhoA-Mediated Cytoskeletal Rearrangements
Source: PLoS Biol. 2010 Aug 24;8(8):e1000457. doi: 10.1371/journal.pbio.1000457 (PMC2927421; doi:10.1371/journal.pbio.1000457)

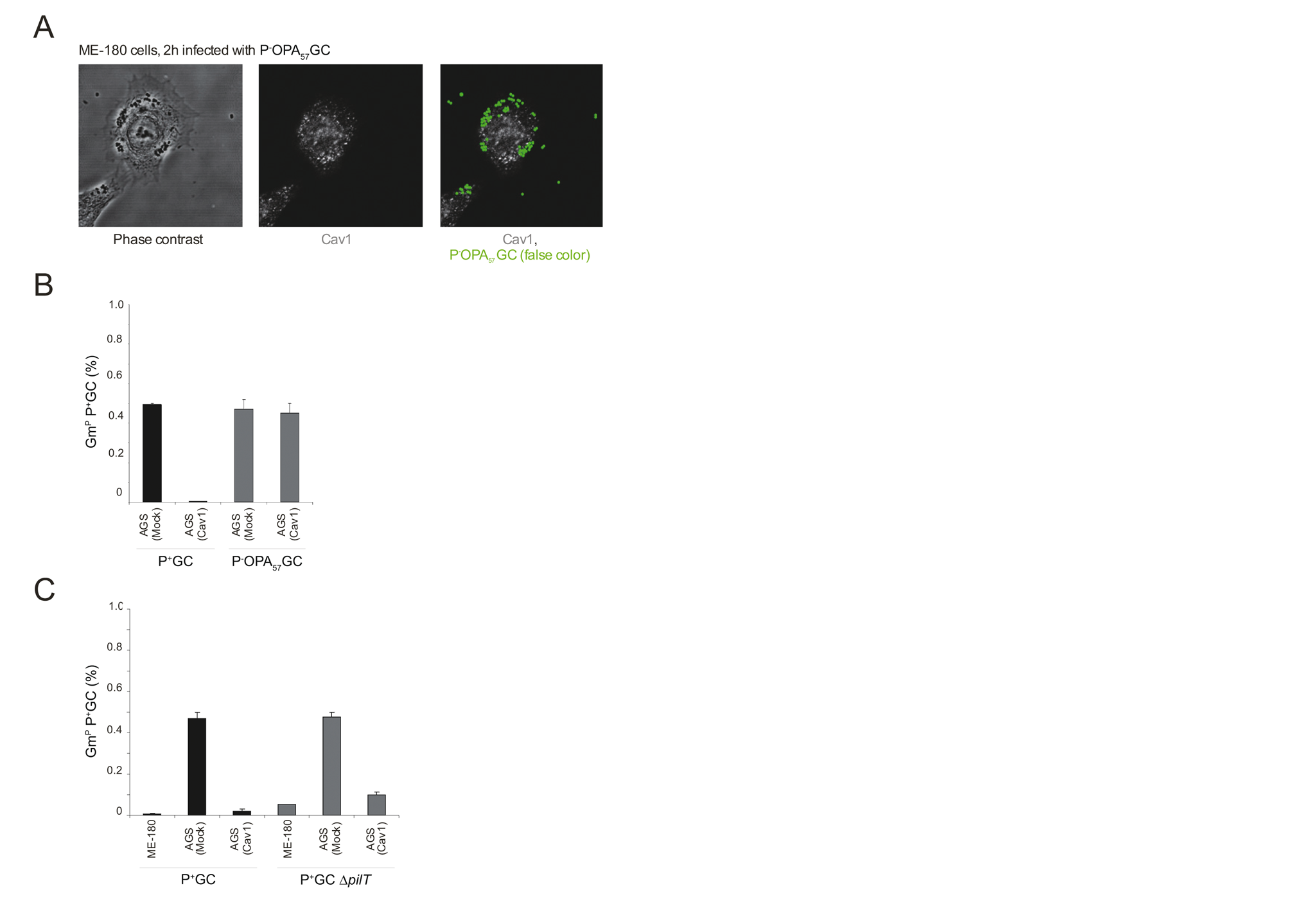

Supplement: Figure S1 — The inhibitory effect of Cav1 on P+GC internalization is pili-specific and independent of pili retraction. (A) Attachment of non-piliated P−Opa57 +GC does not induce Cav1 (white, middle panel) recruitment 2 h post-infection. (B) Expression of Cav1 in AGS cells does not inhibit P−Opa57 +GC internalization. Mock-transfected AGS cells and AGS-Cav1 cells were infected with P+GC or P−Opa57 +GC. (C) Cav1 inhibits uptake of the P+GCΔpilT mutant. AGS-Mock and AGS-Cav1 cells were infected with P+GC or the P+GCΔpilT mutant. Gentamicin protection assays were performed 2 h post-infection. Intracellular gentamicin protected (GmP) bacteria were determined as a percentage of total cell-associated bacteria, which were comparable for the different host cells. Experiments were performed in triplicate. Error bars indicate mean ± standard deviation. (0.36 MB TIF) [file pbio.1000457.s001.tif]

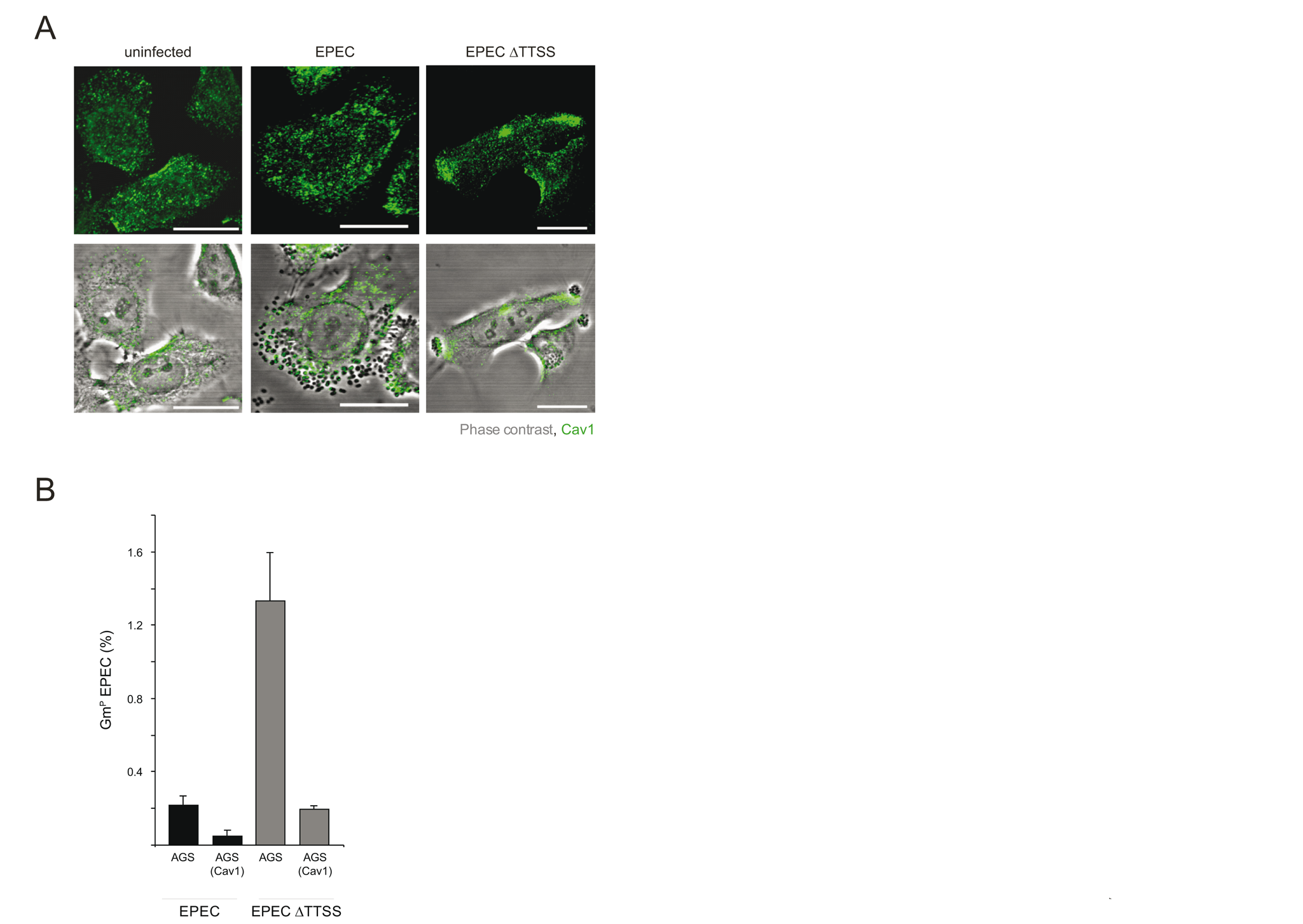

Supplement: Figure S2 — Tfp-producing EPEC induce Cav1 accumulation and prevent host cell entry. (A) ME-180 cells were infected with pre-activated cultures of wild-type EPEC strain E2348/69 and EPEC 2348/69 CVD452, a type III secretion system (TTSS) defective mutant, for 2 h. Endogenous Cav1 (green) is recruited to attachment sites of the microcolony-forming mutant, whereas the TTSS-preactivated wild type adheres dispersed and does not trigger Cav1 recruitment. (B) AGS and AGS-Cav1 cells were infected with E2248/69 wild type and TTSS CVD452 mutant EPEC. Intracellular gentamicin protected (GmP) bacteria were determined as a percentage of total cell-associated bacteria. Experiments were performed in triplicate. Error bars indicate mean ± standard deviation. (0.89 MB TIF) [file pbio.1000457.s002.tif]

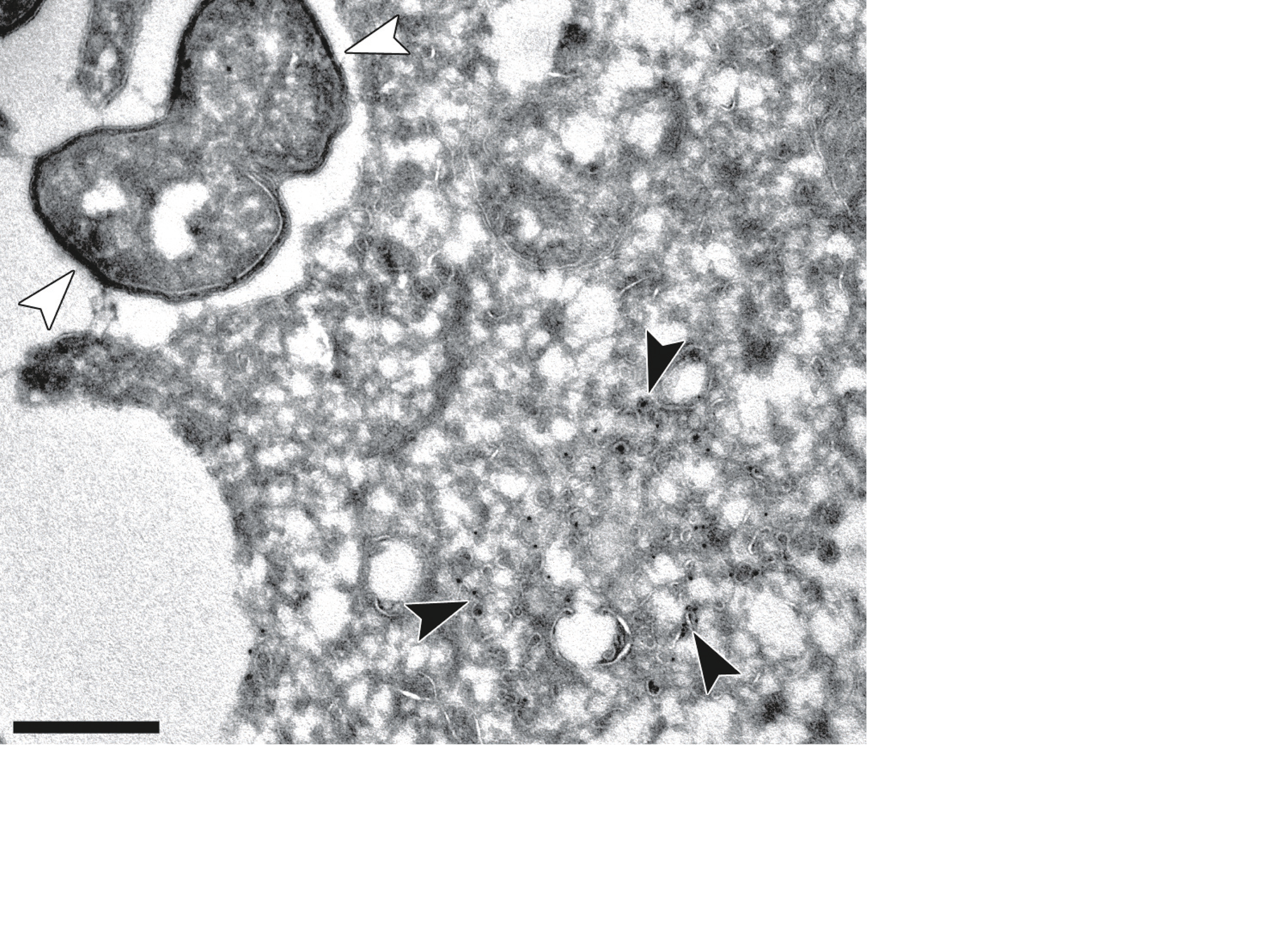

Supplement: Figure S3 — Immunogold labeling of Cav1 in ME-180 cells after infection with P+GC. Cav1 is 6-nm-gold-labeled (black arrows). P+GC are observed as diplococci attached to the cell membrane (white arrows). Scale bar: 500 nm. (3.63 MB TIF) [file pbio.1000457.s003.tif]

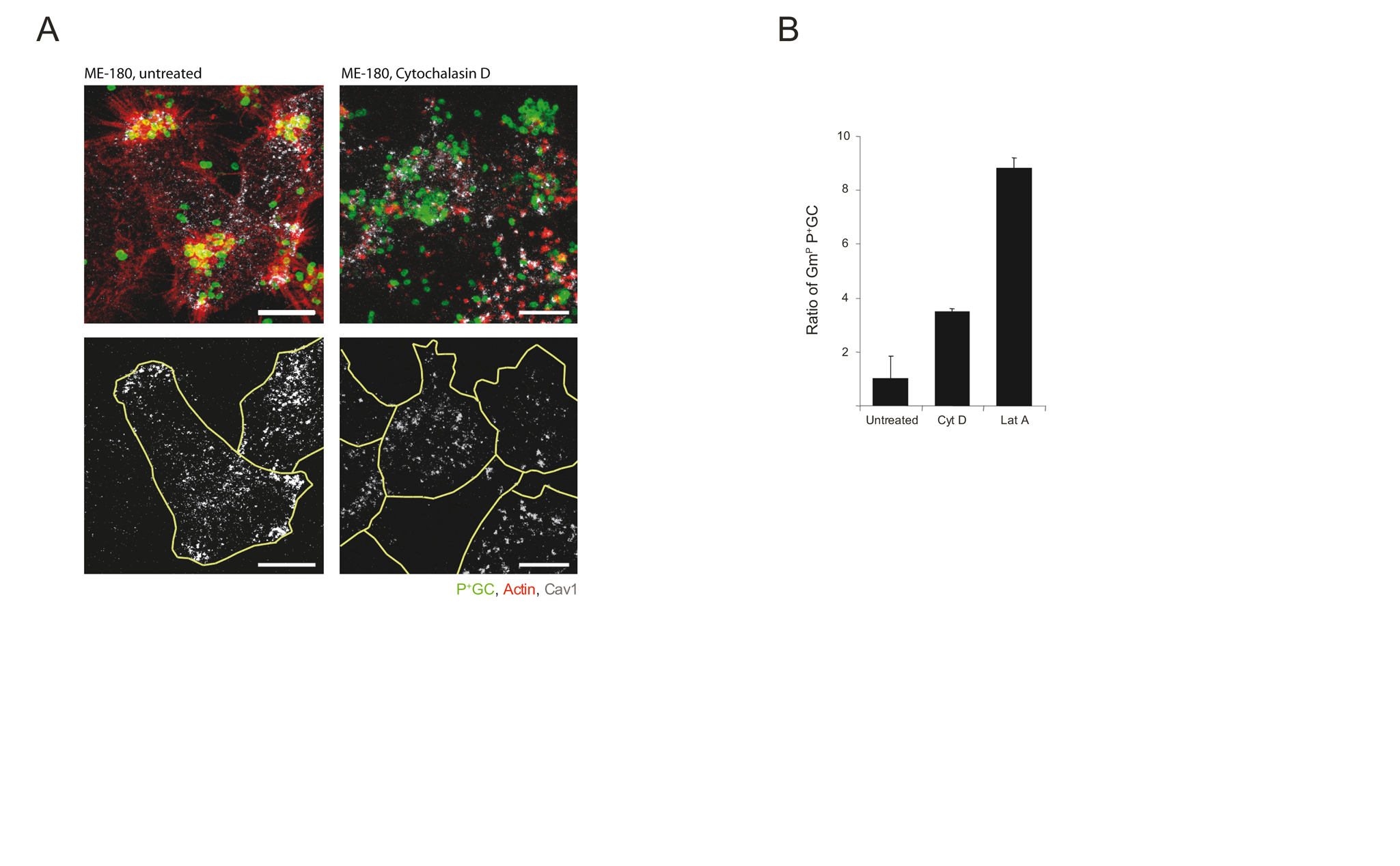

Supplement: Figure S4 — Depolymerization of F-actin induces bacterial internalization and impedes Cav1 recruitment. (A) Disruption of F-actin filaments (red) with cytochalasin D (CytD) prevents Cav1 (white, lower panel) recruitment to P+GC attachment sites (green). Cellular borders are represented as yellow outlines. Scale bars: 20 µm. (B) ME-180 cells were treated with Cyt D or latrunculin A (Lat A), which disrupt actin filaments. Gentamicin protection assay was performed 2 h post-infection. Intracellular GmP bacteria were determined as a percentage of total cell-associated bacteria. Ratio of GmP P+GC calculated relative to untreated control. Experiments were performed in triplicate. Error bars indicate mean ± standard deviation. (1.09 MB TIF) [file pbio.1000457.s004.tif]

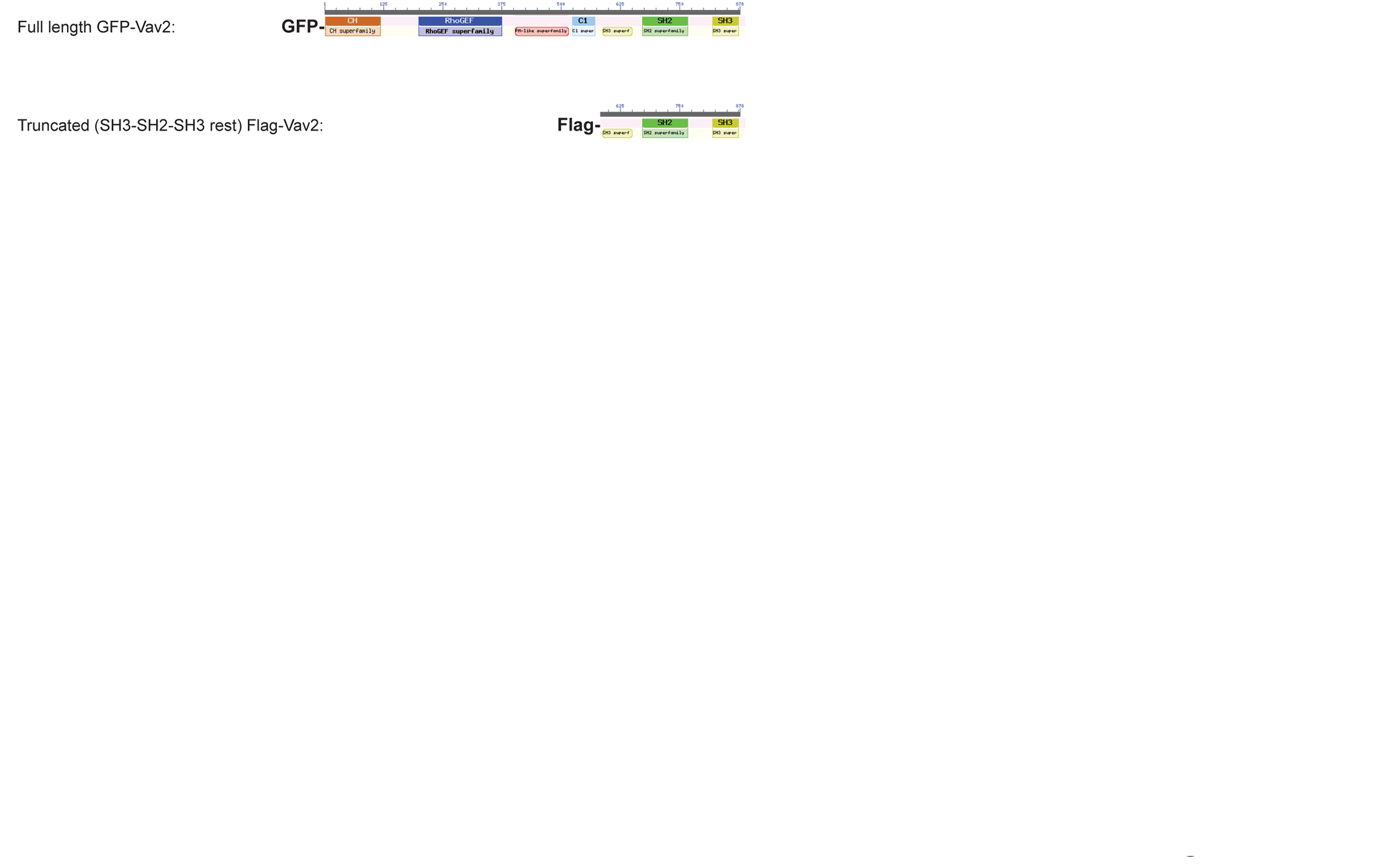

Supplement: Figure S5 — (A) shRNA-mediated downregulation of PLCγ1 in ME-180 cells does not result in P+GC internalization. Only extracellular bacteria (yellow-green) are detected in ME-180 shPLCγ1 cells (upper right panels) and ME-180 shLuciferase control cells (upper left panels). Efficiency of PLCγ1 knockdown in ME-180 cells after lentiviral transduction of luciferase (control) or PLCγ1 shRNA constructs (lower panel). Scale bar: 20 µm. (B) Knockdown of RhoA but not Rac1 or Cdc42 in ME-180 results in P+GC internalization. Intracellular bacteria (red) are detected in siRhoA-treated cells (upper panel, lower right image), whereas only extracellular bacteria (yellow-green) are detected in siCdc42-treated (upper right image), siRac1-treated (lower left image), and siMock-treated cells (upper left image). Knockdown efficiencies of Cdc42, Rac1, and RhoA after siRNA treatment (lower panel; see also Table S2). Scale bar: 20 µm. Data are representative of three independent experiments. (1.15 MB TIF) [file pbio.1000457.s005.tif]

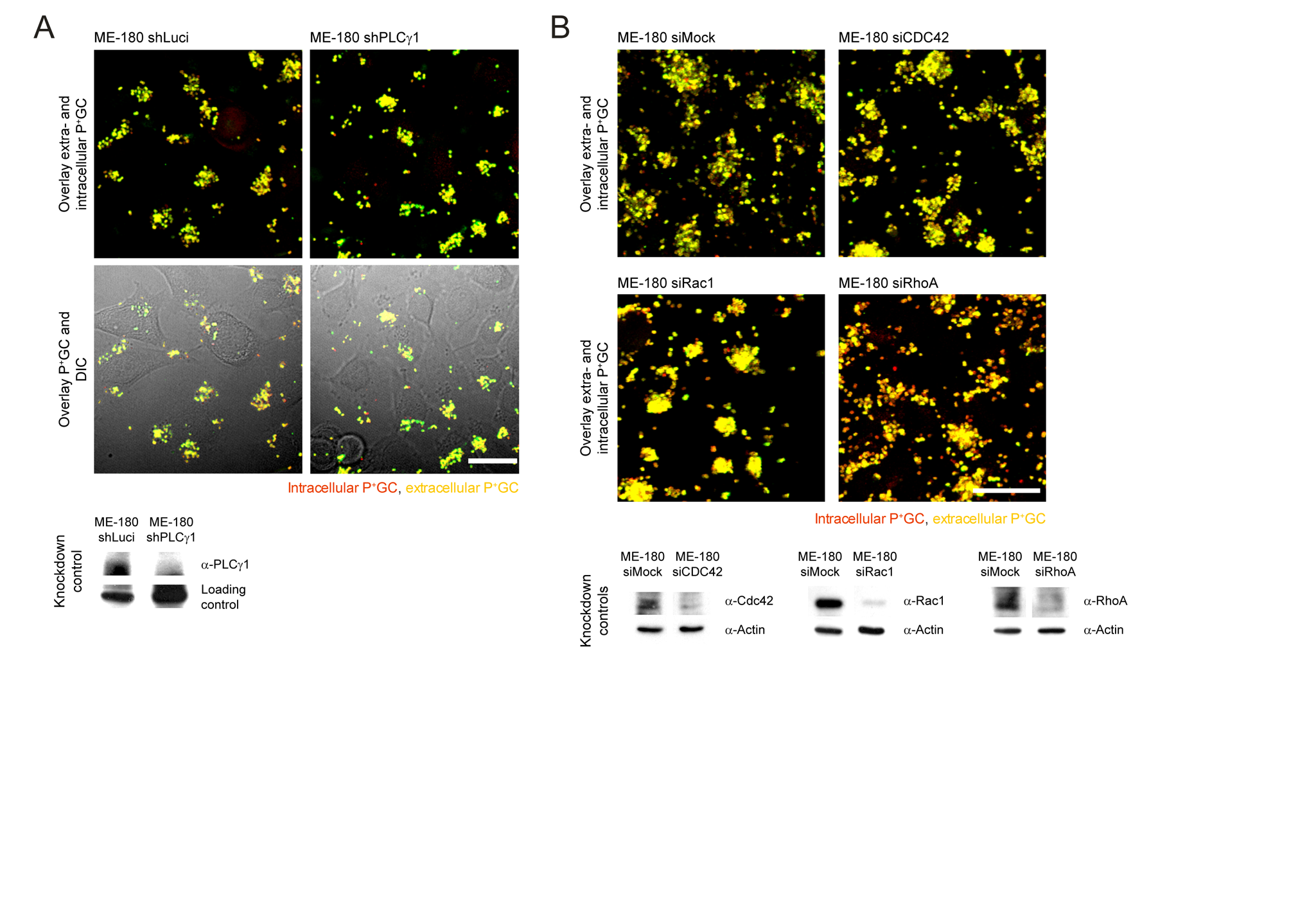

Supplement: Figure S6 — Vav2 constructs used in this work. Full-length GFP-Vav2 cloned into pEGFP-C2 (upper panel) and truncated Vav2 cloned into pcDNA3.FLAG (lower panel). Truncated Vav2 only possesses the C-terminal SH3-SH2-SH3 domains of Vav2. (0.17 MB TIF) [file pbio.1000457.s006.tif]

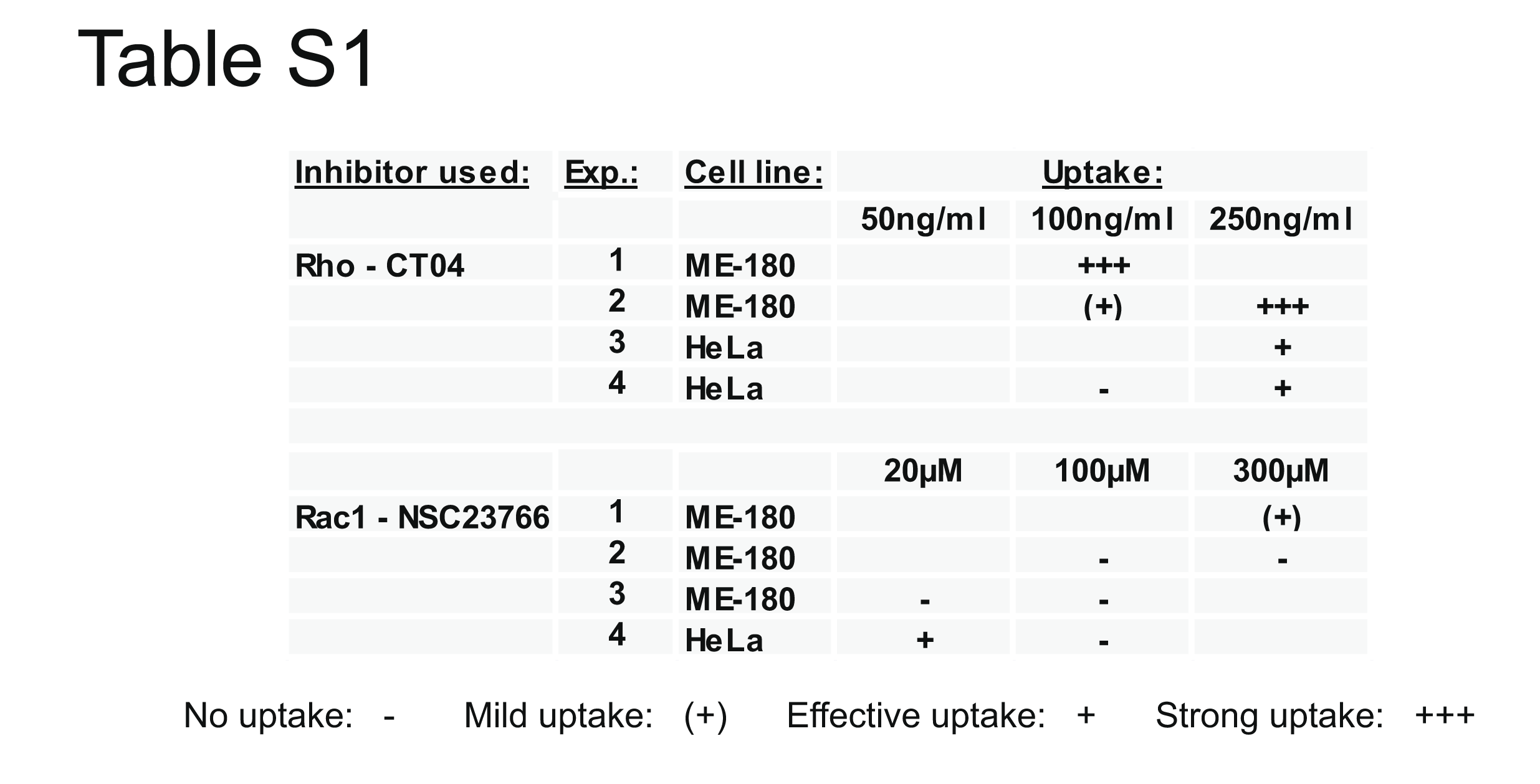

Supplement: Table S1 — Synopsis of Rho- and Rac1-inhibitor experiments shows the importance of Rho to prevent P+GC uptake in different cell lines. The results of eight experiments are summarized here. CT04 exhibited dose-dependent uptake effects in ME-180 as well as HeLa cells in all experiments, whereas, in general, NSC23766 did not impact P+GC internalization. To determine uptake rate—i.e., mild, (+); effective, +; strong, +++—25 or more image stacks of treated and untreated cells per experiment were analyzed for bacterial uptake and cell survival. (0.16 MB TIF) [file pbio.1000457.s007.tif]

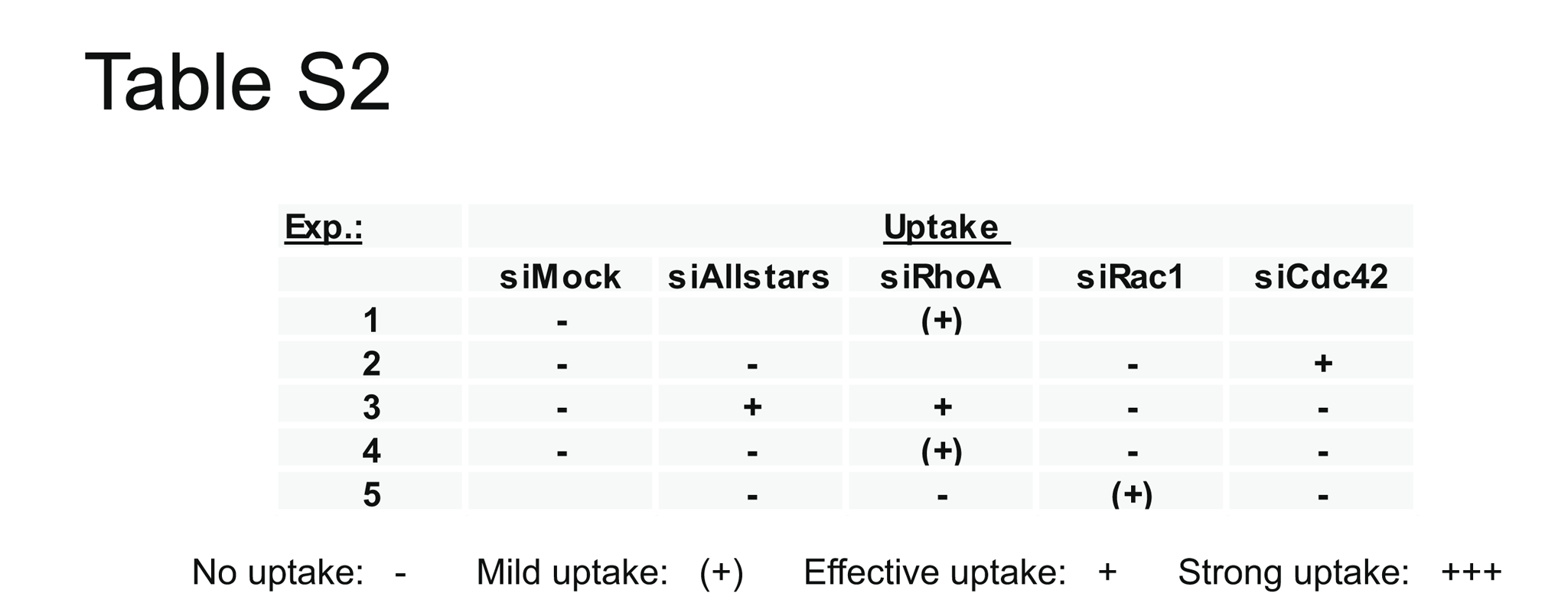

Supplement: Table S2 — Synopsis of siRNA-mediated knockdown experiments of small GTPases Cdc42, Rac1, RhoA underscores the importance of RhoA for preventing P+GC uptake. Results of five experiments are summarized here. In three out of four experiments knockdown of RhoA in ME-180 cells led to increased uptake of P+GC. By contrast, internalized bacteria were detected in only one out of four experiments after siRNA mediated downregulation of Cdc42 and Rac1. To determine uptake rate—i.e., mild, (+); effective, +; strong, +++—25 or more image stacks of treated and untreated cells per experiment were analyzed for bacterial uptake and cell survival. (0.18 MB TIF) [file pbio.1000457.s008.tif]
